# N-glycoproteomic analyses of human intestinal enteroids, varying in histo-blood group geno- and phenotypes, reveal a wide repertoire of fucosylated glycoproteins

**DOI:** 10.1093/glycob/cwae029

**Published:** 2024-04-09

**Authors:** Jonas Nilsson, Inga Rimkute, Carina Sihlbom, Victoria R Tenge, Shih-Ching Lin, Robert L Atmar, Mary K Estes, Göran Larson

**Affiliations:** Department of Laboratory Medicine, Institute of Biomedicine, University of Gothenburg, Sahlgrenska University Hospital, Bruna Stråket 16, SE 413 45, Gothenburg, Sweden; Department of Clinical Chemistry, Region Västra Götaland, Sahlgrenska University Hospital, Bruna Stråket 16, SE 413 45, Gothenburg, Sweden; Proteomics Core Facilities, Sahlgrenska Academy, University of Gothenburg, Medicinaregatan 9E, SE 413 90, Gothenburg, Sweden; Department of Laboratory Medicine, Institute of Biomedicine, University of Gothenburg, Sahlgrenska University Hospital, Bruna Stråket 16, SE 413 45, Gothenburg, Sweden; Department of Microbiology and Immunology, Institute of Biomedicine, University of Gothenburg, Medicinaregatan 7A, SE 413 90, Gothenburg, Sweden; Proteomics Core Facilities, Sahlgrenska Academy, University of Gothenburg, Medicinaregatan 9E, SE 413 90, Gothenburg, Sweden; Department of Molecular Virology, Baylor College School of Medicine, One Baylor Plaza, Houston, TX 770 30, United States; Department of Molecular Virology, Baylor College School of Medicine, One Baylor Plaza, Houston, TX 770 30, United States; Present address: Department of Medicine, Washington University in St. Louis, St. Louis, MO, United States; Department of Molecular Virology, Baylor College School of Medicine, One Baylor Plaza, Houston, TX 770 30, United States; Department of Medicine, Baylor College of Medicine, One Baylor Plaza, Houston, TX 770 30, United States; Department of Molecular Virology, Baylor College School of Medicine, One Baylor Plaza, Houston, TX 770 30, United States; Department of Medicine, Baylor College of Medicine, One Baylor Plaza, Houston, TX 770 30, United States; Department of Laboratory Medicine, Institute of Biomedicine, University of Gothenburg, Sahlgrenska University Hospital, Bruna Stråket 16, SE 413 45, Gothenburg, Sweden; Department of Clinical Chemistry, Region Västra Götaland, Sahlgrenska University Hospital, Bruna Stråket 16, SE 413 45, Gothenburg, Sweden

**Keywords:** glycoproteomics, glycosyltransferases, histo-blood group antigens, human intestinal enteroids, human norovirus

## Abstract

Human noroviruses, globally the main cause of viral gastroenteritis, show strain specific affinity for histo-blood group antigens (HBGA) and can successfully be propagated ex vivo in human intestinal enteroids (HIEs). HIEs established from jejunal stem cells of individuals with different ABO, Lewis and secretor geno- and phenotypes, show varying susceptibility to such infections. Using bottom-up glycoproteomic approaches we have defined and compared the *N*-linked glycans of glycoproteins of seven jejunal HIEs. Membrane proteins were extracted, trypsin digested, and glycopeptides enriched by hydrophilic interaction liquid chromatography and analyzed by nanoLC-MS/MS. The Byonic software was used for glycopeptide identification followed by hands-on verifications and interpretations. Glycan structures and attachment sites were identified from MS^2^ spectra obtained by higher-energy collision dissociation through analysis of diagnostic saccharide oxonium ions (B-ions), stepwise glycosidic fragmentation of the glycans (Y-ions), and peptide sequence ions (b- and y-ions). Altogether 694 unique glycopeptides from 93 glycoproteins were identified. The N-glycans encompassed pauci- and oligomannose, hybrid- and complex-type structures. Notably, polyfucosylated HBGA-containing glycopeptides of the four glycoproteins tetraspanin-8, carcinoembryonic antigen-related cell adhesion molecule 5, sucrose-isomaltase and aminopeptidase N were especially prominent and were characterized in detail and related to donor ABO, Lewis and secretor types of each HIE. Virtually no sialylated N-glycans were identified for these glycoproteins suggesting that terminal sialylation was infrequent compared to fucosylation and HBGA biosynthesis. This approach gives unique site-specific information on the structural complexity of N-linked glycans of glycoproteins of human HIEs and provides a platform for future studies on the role of host glycoproteins in gastrointestinal infectious diseases.

## Introduction

Human norovirus (HuNoV) is estimated to globally cause 680 million cases and approximately 200,000 deaths in acute gastroenteritis each year. After the successful introduction of rotavirus vaccines, HuNoV is now the dominating cause of viral gastroenteritis worldwide (Patel et al. 2008; Hemming et al. 2013; Payne et al. 2013; Becker-Dreps et al. 2014; Bucardo et al. 2014). Noroviruses are classified into ten different genogroups, five of which contain human pathogens, and are further divided into forty-nine genotypes and then into NoV variants (Chhabra et al. 2018; Chhabra et al. 2019; Tenge et al. 2021).

Calicivirus genera, to which the norovirus belongs, show unique species, tissue and cell tropism and give widely different pathologies in the different species and tissues affected (Barron et al. 2011; Abrantes et al. 2012; Neimanis et al. 2018). For HuNoV, the target cells are found in the epithelium of the small intestine (Agus et al. 1973; Schreiber et al. 1973; Schreiber et al. 1974; Dolin et al. 1975; Karandikar et al. 2016; Green et al. 2020) and the symptoms are typically compulsory diarrhea and vomiting, but also commonly fever, headache, abdominal cramps, fatigue and malaise. The epithelia of the human intestines are notoriously difficult to study, but after many years of attempts to culture HuNoV in animals or in *in vitro* cell cultures, a breakthrough was achieved when it was shown that the virus can propagate *ex vivo* in human intestinal enteroids (HIEs) (Ettayebi et al. 2016; Haga et al. 2020). The enteroid cultures were established from stem cells of small intestinal (jejunal) biopsies of individual patients and showed varying susceptibility to HuNoV infection related to their histo-blood group antigen (HBGA) status, particularly to secretor *FUT*2 status (Table 1 and Supplementary Fig. S1), to the HuNoV genotype and strain, and to the presence or absence of bile acids in the inocula (Ettayebi et al. 2016; Haga et al. 2020; Ettayebi et al. 2021). These characteristics confirmed what had been described in the literature from clinical outbreak and challenge studies and underscore this *ex vivo* system as an appropriate model for molecular studies of the HuNoV-host cell infection. A remarkable finding was the ability of such HIEs to support replication also of other previously noncultivatable pathogens such as cryptosporidium (Heo et al. 2018; Wilke et al. 2019) and human sapovirus (Euller-Nicolas et al. 2023).

**Table 1:** HBGA geno- and phenotype characteristics of 7 HIEs established from human jejunal biopsies and analyzed for membrane associated *N*-glycoproteins and susceptibility to HuNoV infection.

HIE ID	Secretor (*FUT2*)		Lewis (*FUT3*) and *ABO*				HuNoV propagation^a^
	Secretor	Genotype	Lewis	Genotype	Genotype	ELISA phenotype	GII.4 SYD propagation
1J	Positive	Se, se^385^	Negative	le^202,314^, le^202,314^	OA	A	+^b^
J2	Positive	Se, se^428^	Positive; Le^b^	Le, Le	OB	B Le^b^	+
J4	Negative	se^428^, se^428^	Positive; Le^a^	Le, le^202,314^	OO	Le^a^	−
J6	Positive	Se, Se	Positive; Le^b^	Le, le^59,508^	OA	A Le^b^	+
J8	Negative	se^428^, se^428^	Positive; Le^a^	Le, le^202,314^	OO	Le^a^	−
J10	Negative	se^428^, se^428^	Negative	le^484,667^, le^59,508^	OA	A^c^	−
J4*FUT2*	Positive	se^428^, se^428^, Se	Positive; Le^b^	Le, le^202,314^	OO	Le^b^	+

Identified mutation sites of *FUT2* and *FUT3* are indicated by smaller case letters and in superscript (e.g. se^385^). Se/se = secretor positive/negative alleles; Le/le = Lewis positive/negative alleles; HIE = human intestinal enteroid; HuNoV = human norovirus.

^a^(Ettayebi et al. 2016; Haga et al. 2020).

^b^Unpublished data.

^c^The A reactivity varied in intensity for different J10 cultures (Rimkute et al. 2020).

A suitable presentation of HBGAs is considered crucial for the susceptibility of intestinal cells to HuNoV infection. However, there are only very few studies reporting on the complete structural characterization of membrane glycoconjugates of human small intestinal cells. Recently, we characterized in structural detail all lipids and glycosphingolipids of the HIE cultures (Rimkute et al. 2020), for which we now present a glycoproteomic analysis. This is the first N-glycoproteomic analysis of human intestinal enteroids in the context of their HBGA status and norovirus susceptibility. Only one early publication by Finne et al., characterized pronase digested glycoproteins of human small intestinal tissues by ^1^H-NMR and mass spectrometry (Finne et al. 1989). Although very detailed in glycan structures, that study did not provide any information as to the carrier glycoproteins nor to the sites of glycan attachments. Studies of released neutral (non-sialylated and non-sulfated) and acidic (sialylated and sulfated) glycans of intestinal mucins of ileum and selected parts of the large intestine of two individuals, both being of the same HBGA phenotype, have recently revealed a decrease in fucosylation and an increase in sialylation towards the distal parts of the human intestinal tract (Robbe et al. 2003; Robbe et al. 2004).

Here we have taken advantage of advanced glycoproteomics methodologies (Chau et al. 2023) to study tryptic glycopeptides from glycoproteins of seven unique HIE cultures. Our data provide clear evidence for a very high structural and site-specific variability of N-linked glycans found in human intestinal glycoproteins of HIEs. If and how any of the HBGAs of these glycoproteins play a role in HuNoV infection will be the subject of further studies.

## Results

### Global characterization of glycoproteins in HIEs

HIEs were prepared from six donors with known HBGA genotypes (Table 1) and one genetically modified secretor positive line (J4*FUT2*), derived by transducing a non-secretor cell line (J4) with a *FUT2* construct (Haga et al. 2020). Total cell membrane preparations, protein digestions with trypsin, HILIC purifications of glycopeptides and finally nanoLC-MS/MS analyses were performed as described in detail in the Materials and methods section. From a total list of 694 confirmed N-glycopeptides identified from 93 N-glycoproteins (Supplementary Table S1), most glycans were of the oligomannose type (Man_5-10_GlcNAc_2_) (Ugonotti et al. 2021). We have here primarily focused on the 257 N-glycopeptide identities, obtained from 24 glycoproteins, that included 1–6 Fuc residues in line with carrying a single core Fuc (12 glycoproteins) or a HBGA structure with or without a core Fuc (12 glycoproteins) (Table 2, Supplementary Table S1). The 24 N-glycoproteins identified in the HIEs represent a broad range of proteins with respect to size and subcellular localization, as well as to cellular functions and associations to various diseases (Supplementary Table S2).

**Table 2:** Glycoproteins, glycopeptides and glycosites carrying N-linked complex-type glycans with HBGA residues defined by the Byonic software and identified in the seven HIEs defined in Table 1.

**Protein**	**Uniprot Accession**	**Sequence**	**Asn Glycosite**	**Present in HIEs**
**Aminopeptidase N**	**P15144**	**AEFNITLIHPK**	**234**	**1J, J2, J6, J8, J10** ^**a**^ **, J4FUT2**
**Aminopeptidase N**	**P15144**	**KLNYTLSQGHR**	**128**	**1J, J2, J4, J8, J10** ^**a**^ **, J4FUT2**
Cadherin-17	Q12864	APKPVEMVENSTDPHPIK	250	J4
Cadherin-17	Q12864	VGNVTAKDPEGLDISYSLR	587	1J, J2, J4FUT2
Carcinoembryonic antigen-related cell adhesion molecule 1	P13688	NQSLPSSER	363	J4
Carcinoembryonic antigen-related cell adhesion molecule 5	P06731	ITPNNNGTYACFVSNLATGR	650	1J, J2, J4, J6
Carcinoembryonic antigen-related cell adhesion molecule 5	P06731	LQLSNDNR	375	1J, J4, J6
Carcinoembryonic antigen-related cell adhesion molecule 5	P06731	LQLSNGNR	197 / 553	1J, J2, J6
**Carcinoembryonic antigen-related cell adhesion molecule 5**	**P06731**	**TLTLFNVTR**	**204 / 560**	**1J, J2, J4, J6, J8** ^**b**^ **, J10** ^a^ ^,^ ^b^
Lysosome-associated membrane glycoprotein 1	P11279	GHTLTLNFTR	103	1J, J2, J4, J6, J10^a^, J4FUT2
Lysosome-associated membrane glycoprotein 1	P11279	LLNINPNK	261	J2, J8
Lysosome-associated membrane glycoprotein 1	P11279	SSCGKENTSDPSLVIAFGR	84	J4, J8
Lysosome-associated membrane glycoprotein 2	P13473	VQPFNVTQGK	35*6*	J2, J8, J10^a^
Mucin-13	Q9H3R2	ADDKFVNVTIVTILAETTSDNEK	284	1J, J4
Olfactomedin-4	Q6UX06	VNLTTNTIAVTQTLPNAAYNNR	352	1J
Polymeric immunoglobulin receptor	P01833	IIEGEPNLKVPGNVTAVLGETLK	469	1J, J2, J4, J8, J4FUT2
Polymeric immunoglobulin receptor	P01833	QIGLYPVLVIDSSGYVNPNYTGR	186	1J, J2, J4
Polymeric immunoglobulin receptor	P01833	VPGNVTAVLGETLK	469	1J, J2
Polymeric immunoglobulin receptor	P01833	VPGNVTAVLGETLKVPCHFPCK	469	J4
Polymeric immunoglobulin receptor	P01833	WNNTGCQALPSQDEGPSK	499	J2, J6
Sucrase-isomaltase, intestinal	P14410	GQFQTFNASYDTINLHVR	1675	J10^a^
**Sucrase-isomaltase, intestinal**	**P14410**	**YIIILDPAISGNETK**	**1303**	**J2, J6, J8, J10, J4FUT2**
**Tetraspanin-8**	**P19075**	**IVNETLYENTK**	**116**	**1J, J2, J4, J6, J8, J10** ^**a**^ **, J4FUT2**
Transmembrane 9 superfamily member 3	Q9HD45	IVDVNLTSEGK	174	J2, J6, J8, J10, J4FUT2

Glycopeptides presented in manuscript or supplementary figures are marked bold. Glycopeptides from 24 unique peptide sequences were carrying HBGA N-glycans, (See also Supplementary Table S2 for a description of the fucosylated glycopeptides identified in the HIEs).

^a^Complex-type glycans without HBGA structure are present in the J10 HIE.

^b^Glycopeptides identified only after manual analysis.

### Characterization of complex-type N-glycans of HIE glycoproteins with respect to ABO, H and Lewis histo-blood group structures

The dominating fucosylated N-glycopeptides will be described in structural detail to illustrate the diversity related to the HBGA epitopes found in the different HIEs. No fucosylated O-linked glycans were confirmed from this analysis, which also included a hands-on inspection of the MS/MS spectra to identify any ions characteristic for O-glycopeptide fragmentation patterns (Halim et al. 2013; Yu et al. 2016), This may be due to the preparation procedure or to the preference of HILIC to enrich for N-glycopeptides having relatively larger glycans compared to typically smaller O-glycans such as core 1 structures. Furthermore, sialylated structures were very scarce and instead, HBGA-related fucosylations completely dominated the complex-type N-glycans.

### Glycoproteomic analysis of complex fucosylated N-glycopeptides

HCD-MS^2^ spectra of oligomannose substituted N-glycopeptides often give the best matching scores in the Byonic analysis due to the typical presence of distinct b- and y-ion series of fragment ions used for peptide sequence identifications (Yu et al. 2016). In this study, glycopeptide identities from fucosylated complex-type N-glycans, with much lower probability scores, were also analyzed provided that the same peptide sequence was identified, typically from an oligomannose glycopeptide with a Byonic score of >200 and a PEP-2D score of <0.001. Such hits were then verified hands-on by the presence of the expected peptide+GlcNAc (HexNAc) ions as diagnostic ions in the MS^2^ spectra and that glycopeptides with identical peptide structures eluted within 0–3 min from each other. Extracted MS^2^ ion chromatograms of selected peptide+HexNAc ions were conveniently used to pinpoint additional glycoforms from the same peptide sequence with the specific purpose of finding and characterizing HBGA containing glycans of the corresponding glycoproteins. Also, many fucosylated complex-type N-glycopeptides with good matching scores (Byonic score > 200, PEP-2D score < 0.001) were identified but were nevertheless manually verified using the above criteria. Glycopeptides of tetraspanin-8 (TSN8), carcinoembryonic antigen-related cell adhesion molecule 5 (CEAM5), sucrose-isomaltase (SUIS) and aminopeptidase N (AMPN), all carrying hybrid- and complex-type N-glycans with HBGA structures were especially prominent and were analyzed in greater detail and compared in-between HIEs varying in their ABO, Lewis and secretor status (Tables 1 and 2, Supplementary Table S1).

### Tetraspanin-8 (TSN8) of J2 HIE

**Fig. 1:**
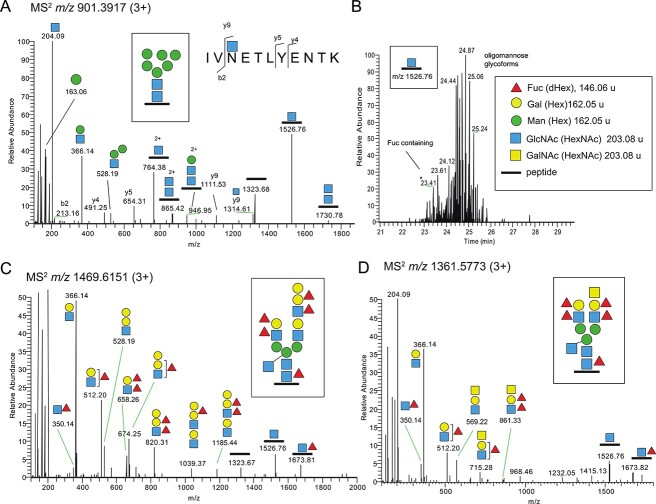
LC-MS/MS of TSN8 N-glycopeptides from J2 and J6 HIEs. A) Example of a Man-6 glycoform including peptide backbone fragmentation. B) Extracted ion chromatogram of the diagnostic peptide+GlcNAc ion (*m/z* 1526.78). C) MS^2^ spectrum of a B Lewis b/y glycoform. D) MS^2^ spectrum of a A Lewis b/y glycoform. Intact glycopeptide structures, corresponding to the MS^1^ precursor ions (*m/z* values), are shown in boxes with symbols as defined in panel B.

A typical example of an oligomannose glycopeptide, peptide sequence IVNETLYENTK, was obtained from TSN8 of the J2 HIE, established from a B Lewis and secretor positive individual (Fig. 1A, Table 1). The Asn-118 attachment site (underlined) was identified and contained the N-glycosylation consensus motif Asn-X-Ser/Thr/Cys (X is not Pro). (The *m/z* values of annotated fragment ions are from the largest isotope peaks, and therefore *m/z* values sometimes differ between spectra by ±1 m/z units for 1+ fragment ions and ± 0.5 m/z units for 2+ fragment ions.) An extracted ion chromatogram (XIC) at the MS^2^ level covering the diagnostic peptide+GlcNAc ion of TSN8 glycopeptides (*m/z* 1526.76) of the J2 HIE preparation clearly indicated the presence of many different glycoforms (Fig. 1B). The ion peaks at m/z 1526.76 were dominated by oligomannose precursor ions in this sample, but also contained many glycopeptides that included Fuc on the terminal GalGlcNAc antennae of complex bi- and triantennary structures. One of the major fucosylated glycoforms of TSN8 in the J2 sample appeared at *m/z* 1469.6151 (z = 3) and eluted at 23.41 min (Fig. 1B and C). This ion corresponds to a Hex_7_HexNAc_6_dHex_5_ glycan on the IVNETLYENTK peptide with a proposed structure of a complex biantennary glycan with a bisecting GlcNAc and a difucosylated Lewis b/y structure on one branch and a B Lewis b/y structure on the other branch (Fig. 1C). The HCD-MS^2^ spectrum included diagnostic Fuc (dHex)-containing oxonium ions (also known as B-ions) at *m/z* 350.14; 512.20; 658.26; 674.25; 820.31; 1039.38, and 1185.44. The identities of HBGA-related oxonium ions are all presented in Table 3. Based on the presence of the *m/z* 512.20 ion, Fuc may be attached to either the terminal Gal or to the subterminal GlcNAc, whereas the presence of an ion at *m/z* 350.14 should be significant for a Fuc attached to GlcNAc, typical for HBGA Lewis structures. However, fucose units are known to migrate between adjacent sugar residues during positive-mode ionization and collisional dissociation, known as fucose migration (Mucha et al. 2018; Lettow et al. 2023; Moge et al. 2023). Therefore, the presence of an ion at *m/z* 350.14 could very well also arise when the Fuc is attached to the neighboring Gal residue. Importantly, possible Fuc migration was not detrimental to the analysis of the identified polyfucosylated TSN8 glycopeptides since the established glycan compositions included presence of two Fuc residues for each terminal GalGlcNAc unit and a fifth Fuc linked to the core GlcNAc.

**Table 3:** Diagnostic oxonium ions found in LC-MS/MS analyses and related to HBGA structures.

**Mass (u)**	**Composition**	**Proposed N-glycan epitope**	**Proposed HBGA identity**
350.1446	HexNAc dHex	FucGlcNAc	Lewis
512.1974	Hex HexNAc dHex	FucGalGlcNAc	H
512.1974	Hex HexNAc dHex	Gal(Fuc)GlcNAc	Lewis a/x
528.1923	Hex_2_ HexNAc	GalGalGlcNAc	-
528.1923	Hex_2_ HexNAc	ManManGlcNAc	-
569.2189	Hex HexNAc_2_	GalNAcGalGlcNAc	-
658.2553	Hex HexNAc dHex_2_	FucGal(Fuc)GlcNAc	Lewis b/y
674.2502	Hex_2_ HexNAc dHex	Gal(Fuc)GalGlcNAc	B
715.2768	Hex HexNAc_2_ dHex	GalNAc(Fuc)GalGlcNAc	A
731.2717	Hex_2_ HexNAc_2_	GalGlcNAcGalGlcNAc	-
820.3081	Hex_2_ HexNAc dHex_2_	Gal(Fuc)Gal(Fuc)GlcNAc	B Lewis b/y
861.3347	Hex HexNAc_2_ dHex_2_	GalNAc(Fuc)Gal(Fuc)GlcNAc	A Lewis b/y
877.3296	Hex_2_ HexNAc_2_ dHex	FucGalGlcNAcGalGlcNAc	H
877.3296	Hex_2_ HexNAc_2_ dHex	Gal(Fuc)GlcNAcGalGlcNAc	Lewis a/x
877.3296	Hex_2_ HexNAc_2_ dHex	GalGlcNAcGal(Fuc)GlcNAc	Lewis a/x
1023.3875	Hex_2_ HexNAc_2_ dHex_2_	FucGal(Fuc)GlcNAcGalGlcNAc	H
1023.3875	Hex_2_ HexNAc_2_ dHex_2_	Gal(Fuc)GlcNAcGal(Fuc)GlcNAc	Lewis a/x
1023.3875	Hex_2_ HexNAc_2_ dHex_2_	FucGal(Fuc)GlcNAcGalGlcNAc	Lewis b/y
1039.3824	Hex_3_ HexNAc_2_ dHex	Gal(Fuc)GalGlcNAcGalGlcNAc	B
1185.4403	Hex_3_ HexNAc_2_ dHex_2_	Gal(Fuc)Gal(Fuc)GlcNAcGalGlcNAc	B Lewis b/y

Notably, we did not observe any ions at *m/z* 350.14 or *m/z* 512.20 for the N-glycopeptides of TSN8 from the J10 HIE, being Lewis negative and non-secretor (Table 1), still having a core Fuc but lacking antennae fucosylation. This indicates that the core α1,6Fuc was not responsible for the presence of the *m/z* 350.14 ion in the polyfucosylated N-glycopeptides, and that fragmentation-induced fucose migration of the core α1,6Fuc to another GlcNAc was not present during the LC-MS/MS conditions used in this study. Additionally, for the J2 fraction, the oxonium ion at *m/z* 658.26 corresponding to a tetrasaccharide with one Fuc attached to each monosaccharide residue of the GalGlcNAc disaccharide was indeed also identified indicating the presence of a HBGA Lewis b/y structure. Moreover, for the J2 fraction, the prominent presence of ions at *m/z* 528.19; 674.25 and 820.31, carrying an additional Hex residue, is most likely due to the presence of a blood group B Lewis b/y type structure (Gal(Fuc)Gal(Fuc)GlcNAc) (Fig. 1C). This interpretation is based on the observation that the ion at *m/z* 528.19 (Hex_2_HexNAc corresponding theoretically to either GalGalGlcNAc, GalGlcNAcMan or ManManGlcNAc) is typically a less intense peak for complex biantennary GalGlcNAc terminated glycoforms as well as for oligomannose glycoforms (Fig. 1A). Indeed, most HexNAc-containing oxonium ions preferentially have a HexNAc, rather than a Hex, at their reducing ends (Yu et al. 2016). Additional support for the B Lewis b/y structure was obtained from the oxonium ions at *m/z* 1039.38 and 1185.44 (Fig. 1C) indicating that the precursor ion included a GalGlcNAc elongation of the complex biantennary structure rather than a triantennary glycoform. Furthermore, the identification of an additional HexNAc residue on the innermost Man, is in line with the presence of a bisecting GlcNAc, seen in the MS^2^ spectra of other TSN8 glycoforms, including a number of hybrid-type structures carrying only Man on one antenna and Fuc modifications of GalGlcNAc on the other antenna (Supplementary Fig. S2).

Thus, the combination of characteristic oxonium ions, glycosidic fragmentations of the glycopeptide including the peptide+GlcNAc (*m/z* 1526.76) and peptide+GlcNAc+Fuc ions (*m/z* 1673.81), and the total mass of the precursor ion collectively pointed to the depicted complex structure (Fig. 1C boxed). This is, however, only one of the theoretically possible structures with identical *m/z* values since, for instance, the GalGlcNAc extension may be on either of the two antennas, and additionally, isobaric glycoforms may co-elute and together contribute to the MS^2^ spectra.

In support of the suggested structure, the complex N-glycopeptides of TSN8 from the J6 HIE, having an ALe^b^ phenotype, typically carried difucosylated structures with a terminal HexNAc on one of the antennae, in line with the GalNAc residue of the ALe^b^ epitope and based on the presence of the *m/z* 569.22; 715.28; and 861.33 oxonium ions (Fig. 1D, Table 3).

**Fig. 2:**
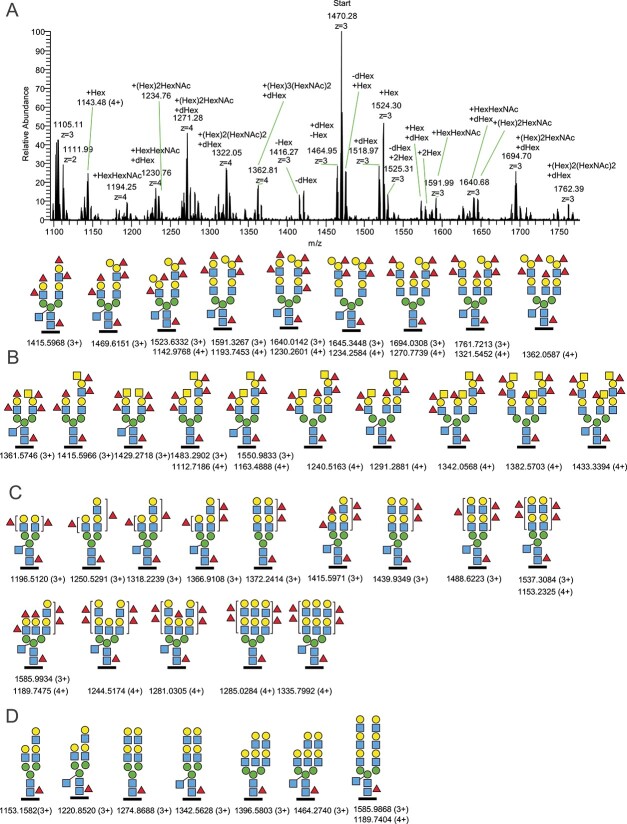
Examples of major glycoforms of TSN8 detected from J2, J6, J8 and J10 HIEs. A) MS^1^ scan of the J2 sample (top) where the ions are related to the prominent ion at *m/z* 1470.28, which is the most intense isotopic peak for this precursor ion; and the proposed glycopeptide structures (bottom) given with four decimals of the monoisotopic masses. B) Example of glycoforms from the J6 HIE; C) the J8 HIE and D) the J10 HIE.

In addition, a range of even more complex TSN8 derived N-glycopeptides eluted separately for the J2 and J6 HIEs (Fig. 2A and B). For the J2 MS^1^ spectrum shown, summed over 23.2–23.6 min, the delta masses of these 3+ and 4+ charged precursor ions equated, in relation to the dominating ion at *m/z* 1470.28, to the tentative structures displayed. For J2, two of these additional precursor ions were subjected to MS^2^ analysis for confirmation (Supplementary Fig. S2D and E); and for J6, three additional precursor ions were subjected to MS^2^ (Supplementary Fig. S3A–C).

### TSN8 of additional HIEs

Since TSN8 has only one consensus N-glycosylation site and this glycopeptide appeared in our LC-MS/MS analysis in so many nicely fragmented glycoforms, we used this glycopeptide to compare the complex-type N-glycans of all HIEs, varying in their HBGA status (Table 1). The TSN8 glycoforms originating from the J2, J6, J8, and J10 cells differed considerably as to the oxonium ion profiles relating to their ABO, Lewis and secretor types (Fig. 2A–D). For instance, the terminal Hex_2_HexNAcFuc_2_ ion (*m/z* 820.31) was only present for the J2 B Lewis and secretor positive cells (Fig. 2C, Supplementary Fig. S2D and E) whereas the corresponding HexHexNAc_2_Fuc_2_ ion (*m/z* 861.33) was exclusively found in J6 A Lewis and secretor positive cells (Fig. 1D, Supplementary Fig. S3A–C). The J8 O Lewis positive secretor negative cells showed, compared to J2 and J6 cells, more of only mono-fucosylated substitutions on the GalGlcNAc extensions (*m/z* 512.20), compatible with H or Lewis a/x structures (Supplementary Fig. S3D–G), but was completely lacking A or B-like HBGAs. However, minor amounts of di-fucosylated terminals corresponding to Lewis b/y structures were also identified in this non-secretor HIE (Supplementary Fig. S3F). Finally, spectra of J10 A Lewis and secretor negative cells did not include any Fuc containing N-glycopeptides on the antennae of any major TSN8 glycopeptides (Fig. 2D), which were all instead terminated with unsubstituted GalGlcNAc structures supporting the α1,2- and α1,3/4-fucosylation deficiencies in these HIE cells. A GalGlcNAc elongation of the antennae was suggested due to the presence of the ion at *m/z* 731.27 corresponding to a GalGlcNAcGalGlcNAc tetrasaccharide and also of a corresponding hexasaccharide ion at *m/z* 1096.40 (Supplementary Fig. S3H and I). The major complex-type N-glycans of the TSN8 derived glycopeptides of all the individual HIEs contained a fucosylated core GlcNAc residue and a bisecting GlcNAc residue.

### Additional analysis of HBGA glycoproteins

In order to compare the patterns of glycosylation of TSN8 with other glycoproteins appearing in the individual HIE preparations, extracted ion chromatograms (XICs) were used for identifying fucosylated oxonium ions originating from glycopeptides. Specifically, the presence of Fuc-containing glycans was searched for using the typical oxonium ions at *m/z* 350.14; 512.20 and 658.26 (Supplementary Fig. S4). The secretor and Lewis positive J2 and J6 HIEs displayed many different N-glycopeptides with mono- and difucosylations of the antennae (*m/z* 512.20 and *m/z* 658.26, Table 3), including fucosylation of the GlcNAc residue (*m/z* 350.14) (Supplementary Fig. S4A and B). The non-secretor J8 and J10 HIEs also displayed N-glycopeptides carrying a monofucosylated terminal; and for J8, typical fucosylation of the GlcNAc was also indicated. However, difucosylation was detected only in very minor amounts for the J8 and was completely absent for the J10 HIE.

Furthermore, XICs of the oxonium ions specific for the HBGA B mono- and difucosyl epitopes at *m/z* 674.25 (Hex_2_HexNAcFuc) and *m/z* 820.31 (Hex_2_HexNAcFuc_2_); and for A epitopes at *m/z* 715.28 (HexHexNAc_2_Fuc) and *m/z* 861.33 (HexHexNAc_2_Fuc_2_) were analyzed for J2, J6, J8 and J10 HIEs (Supplementary Fig. S5). We selectively detected B epitopes in the J2 HIE and A epitopes in the J6 HIE but did not detect either of those in the J8 or J10 HIEs, which matches well with the HBGA ABO secretor and Lewis genotypes of these HIEs (Table 1).

**Fig. 3:**
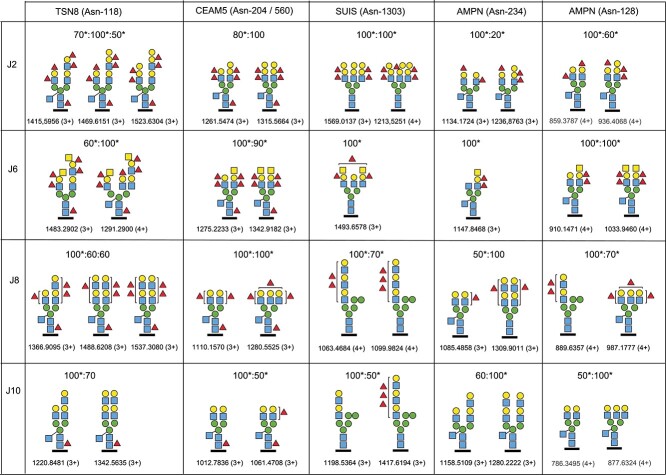
Major complex- and hybrid-type glycopeptides identified from TSN8, CEAM5, SUIS and AMPN in the J2, J6, J8 and J10 HIEs. The Asn glycosylation sites are indicated on top together with the abbreviated glycoprotein names. Two sites from AMPN are presented. The relative abundance, *m/z* and charge of precursor ions are indicated. ^*^The MS^2^ spectra of these glycopeptides are presented in Supplementary Figs S2, S3, and S6–S9.

Additionally, we identified three other major highly fucosylated glycoproteins that were selected for further analyses, mainly due to their considerable structural and functional differences and the prominent presence of fucosylated oxonium ions and glycopeptide identities across the different HIEs; carcinoembryonic antigen-related cell adhesion molecule 5 (CEAM5), sucrose-isomaltase (SUIS) and aminopeptidase N (AMPN) (Table 2, Supplementary Table S1). TSN8 is a multi-pass membrane protein with a mass of only 26 kDa and only one N-glycosylation site, whereas CEAM5 is a plasma membrane associated GPI-anchored glycoprotein with a MW of 180 kDa and a peptide mass of 77 kDa. The CEAM5 sequence contains 28 potential N-glycosylation sites. We identified five glycosylation sites of CEAM5; one site having only oligomannose (Asn-508) and four sites having both oligomannose and complex-type glycans (Asn-197/Asn-553, Asn-204/Asn-560, Asn-375, and Asn-650). Further, we identified six N-glycosylation sites of SUIS, which is synthesized as a single pass type 2 membrane glycoprotein cleaved by pancreatic proteases, and which appears in the intestinal lumen as a mixed heterodimer with the two enzymatic activities non-covalently complexed together. Its protein mass is 209 kDa covering 1827 amino acids. SUIS harbors 18 potential N-linked glycans. In this study, we identified oligomannose glycans at Asn-437, Asn-823, Asn-1354, Asn-1675 and Asn-1763, whereas complex-type N-glycans carrying HBGAs were found at Asn-1303 and Asn-1675. Finally, AMPN is a single pass type 2 membrane glycoprotein with N-terminal protease activity, mass 110 kDa and with 10 potential N-glycosylation sites. We identified two of these sites (Asn-265 and Asn-818) carrying only oligomannose and two sites (Asn-128 and Asn-234) carrying oligomannose and complex-type glycans including HBGAs. The major HBGA and related N-glycopeptides identified from these proteins originating from the J2, J6, J8 and J10 HIEs are presented in Fig. 3. The relative abundances of the 1-3 most prominent HBGA and related N-glycopeptide precursor ions were measured and are presented for each HIE. Also, all Byonic-identified N-glycopeptides are presented in Supplementary Table S1.

### HBGA N-glycopeptides of CEAM5

Complex-type N-glycopeptides of CEAM5 with the sequence TLTLFNVTR, containing the Asn-204/560 N-glycosylation sites, were particularly abundant in the J6 HIE, but also present in the 1J, J2, J4, J8 and J10 HIEs (Table 2, Fig. 3, Supplementary Figs S4–S6). The HBGA epitopes of CEAM5 were largely similar to the ones found for TSN8 from the same individual HIEs. The presence of peptide+HexNAc_3_Hex ions at *m/z* 1835.90 and *m/z* 1836.90 established the presence of bisecting GlcNAc. Furthermore, an ion corresponding to *^0,2^X* cleavage of the N-glycosidically linked GlcNAc residue was identified at *m/z* 1147.65, that was generally beneficial for assigning the peptide ion/peptide+HexNAc ion pair. Di-fucosylation of the GalGlcNAc antennae was again detected for the two secretor and Lewis positive HIEs, i.e. J2 and J6, whereas mono- or no fucosylation of the antennae was detected for the two non-secretor HIEs, i.e. J8 and J10.

### HBGA N-glycopeptides of sucrase-isomaltase (SUIS)

Although TSN8- and CEAM5-derived glycopeptides dominated the XICs obtained for the oxonium ion at *m/z* 512.20 in J2 and J6 HIEs, another cluster of precursor ions was particularly abundant from the glycopeptide YIIILDPAISGNETK, modified at Asn-1303 and derived from SUIS of the J8 and J10 HIEs (Supplementary Fig. S4). These two HIEs exhibited glycoforms of which several had hybrid-type structures (Fig. 3, Supplementary Fig. S7). Glycoforms of this glycopeptide but with complex-type HBGA B- and A-like epitopes were found only for the J2 and J6 HIEs (Supplementary Fig. S7).

### HBGA N-glycopeptides of aminopeptidase N (AMPN)

Using the XICs of diagnostic ions *m/z* 512.20, 350.14; 658.26; 674.25; 820.31; 715.28 and 861.33 AMPN was identified in two clusters of fucosylated glycopeptides. Both of these two glycopeptides, AEFNITLIHPK and KLNYTLSQGHR, appeared with oligomannose, hybrid- and complex-type modifications in the HIEs and their fucosylation patterns of the dominating complex-type N-glycans were principally similar to those of the other three glycoproteins for all of the HIEs (Fig. 3, Supplementary Figs S8 and S9). Difucosylated HBGA B and A epitopes were only found in J2 and J6 HIE preparations, respectively; monofucosylated epitopes were dominating in J8 and non-fucosylated glycans were dominating in the J10 preparations. For these glycopeptides of AMPN, core fucosylations were absent and bi-antennary structures, including bisecting GlcNAc, were dominating. The glycoforms of the J8 HIE were annotated to have tri-antennary structures since the fragment ion at *m/z* 731.27, or its fucosylated variants (Table 3), diagnostic for elongated antennae, were not observed. For the Asn-234 site we identified a glycopeptide carrying a singly sialylated glycan from the J10 HIE. This finding indicates that terminal sialylation of N-glycans is indeed very rare, although not totally absent, from these HIEs.

### Characterization of complex-type N-glycans of J4, J4FUT2 and 1J HIE glycoproteins

**Fig. 4:**
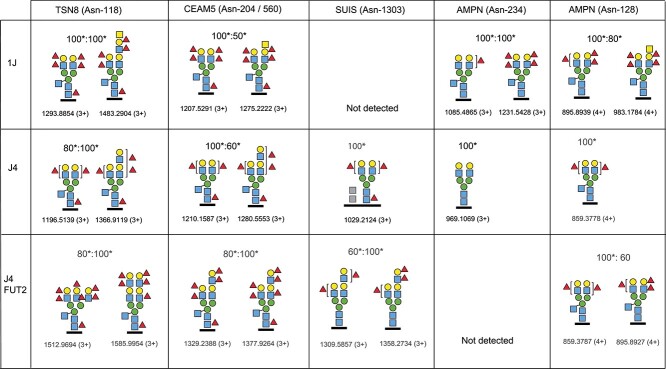
Major complex-type glycopeptides identified from TSN8, CEAM5, SUIS and AMPN in the 1J, J4, and J4FUT2 HIEs. The Asn glycosylation sites are indicated as in Fig. 3. Two sites from AMPN are presented. The relative abundance, *m/z* and charge of precursor ions are indicated. ^*^The MS^2^ spectra of these glycopeptides are presented in Supplementary Figs S10–S13. Grey square, HexNAc of unknown position and attachment site.

Since the J4 HIE was established from an O Lewis positive secretor negative individual we transduced this HIE with a lentivirus vector carrying a functional secretor allele into the J4*FUT2* HIE to specifically study the effect of the added fucosyltransferase (Haga et al. 2020). This transduction changed the HIE from a HuNoV infection-resistant cell line to a line susceptible to GII.4 Sydney infection (Table 1). The HBGA patterns of the glycoproteins studied in detail for the other native HIEs (TSN8, CEAM5, SUIS and AMPN) now showed a site-specific increase of N-glycans with difucosylated rather than monofucosylated antennae compared to the original J4 (Fig. 4, Supplementary Figs S10–S12). This appearance of Lewis b/y structures rather than Lewis a/x (or H) structures is nicely compatible with the added activity of the *FUT2* secretor α1,2-fucosyltransferase.

The presence of difucosylated Lewis structures in the 1J HIE is noteworthy (Fig. 4, Supplementary Figs S10, S11, and S13) since this culture was genotyped as A Lewis negative (le^202,314^, le^202,314^) secretor positive and was thus not expected to synthesize ALe^b^ or Le^b^ epitopes, unless there was an additional expression of another α1,4-fucosyltransferase, such as *FUT5*, in these cells. In a transcriptomic dataset from the J2 HIE there were, however, no transcripts of *FUT5* detected in these cells but low expression of *FUT1* and strong expression of *FUT4* and *FUT6*, both coding for α1,3-fucosyltransferases (Supplementary Table S3). Thus, we interpret this as an indication that the difucosylated HBGA epitopes detected in 1J HIEs are the type 2 chain ALe^y^ and Le^y^ epitopes.

### Sialylated N-glycopeptides of HIE preparations

The first Byonic analysis of the 1J, J2, J4, J6 and J4FUT2 HIEs identified a number of sialylated N-glycopeptides, primarily from laminins (Supplementary Tables S1 and S4). However, since both Neu5Ac and Neu5Gc glycoforms were identified, and Neu5Gc is usually not present in human samples, we anticipated that these identities originated mainly from the mouse-derived Matrigel product used for the cell culture. Indeed, mouse database searches identified those sialylated glycopeptide hits and also additional N-glycopeptide identities with unique mouse sequences (Supplementary Table S4, Supplementary Fig. S14). Also, by applying our glycoproteomics protocol directly to the Matrigel product itself we found very similar N-glycopeptide profiles (not shown). Thus, the glycopeptide identities from laminins were very likely to arise from the Matrigel remains in the HIE preparations. From these data we conclude that our HILIC methodology enriches also sialylated N-glycopeptides, such as the one described for AMPN of the J10 HIE. However, another possibility with regards to the lack of Neu5Ac glycopeptide identities is that one Neu5Ac (291 Da) may be mistaken for two Fuc residues (292 Da) in combination with an allowed −1 Da assignment of the monoisotopic mass in the Byonic analysis (Chau et al. 2023). Therefore, we conducted a second Byonic analysis using 96 N-glycan compositions including 1–4 Neu5Ac residues, but still no Neu5Ac N-glycopeptides originating from HIE membrane glycoproteins were identified in any of the samples (Supplementary Tables S5 and S6). Further, in our manual analysis of the MS^2^ spectra we could not detect any presence of the Neu5Ac specific ion at *m/z* 274.09 in combination with the peptide+HexNAc ion for any of the HBGA related glycopeptides, except for the example of an AMPN glycopeptide in J10 (Supplementary Figs S8 and S15). Thus, no Neu5Ac glycoforms were detectable, and although a relatively wide isolation window was used (5 m/z units) this did not result in any detectable ions at *m/z* 274 or *m/z* 290 due to interfering co-fragmentation of HIE-derived and Matrigel-derived glycopeptides. Altogether, these results underscore our general finding of very low levels of sialylated N-glycoproteins in these jejunal HIEs.

## Discussion

This work represents the first N-glycoproteomic analysis of human intestinal enteroids in the context of their HBGA status and norovirus susceptibility. The enteroids were established from stem cells of biopsies taken from the jejunum of six individuals varying in their ABO, Lewis and secretor geno- and phenotypes. Special attention was given to compare the HBGA structures and their sites of attachment on carrier proteins and for this purpose we selected four of the most abundant intestinal glycoproteins carrying N*-*linked hybrid- and complex-type N-glycans. These four glycoproteins were experimentally found in at least six of the seven HIEs analyzed, and represent structurally very diverse proteins that in the intestinal tissue are embedded in the plasma membrane, extend out from the plasma membrane of the epithelial cells, or are shed from the epithelial cells into the intestinal lumen.

We screened the MS-data from HIEs for typical HBGA epitopes of these four N-glycoproteins using diagnostic MS^2^ fragment ions and used extracted ion chromatograms to get an HBGA profile of each HIE. We observed distinct fragmentation patterns of glycopeptides into oxonium ions (B-ions), glycosidic fragments (Y-ions) and peptide backbone fragments at high mass accuracy providing an efficient structural characterization. The Byonic approach, fragmentation profiles and diagnostic oxonium ion identities presented here (Table 3) should all be valuable for glycoproteomic studies of polyfucosylated glycopeptides. Our analytical approach thus includes 1) a composite identification workflow using FDR, Byonic score, PEP-2D score and manual verifications to provide for a stringent glycopeptide identification methodology; 2) use of extracted ion chromatograms of selected oxonium ions to qualitatively assay unique glycan HBGA epitopes (Supplementary Figs S4 and S5), 3) identification and use of diagnostic oxonium ions, in particular *m/z* 731.27 (Hex_2_HexNAc_2_) and its fucosylated variants, for finding elongations of the N-glycan antennae, and *m/z* 658.26 (HexHexNAcdHex_2_) for identification of Lewis b/y epitopes, 4) search for the presence of fragment ions containing the peptide plus a core tetrasaccharide (HexHexNAc_3_) and related ions for the identification of a bisecting GlcNAc residue, and 5) use of the diagnostic *^0,2^X* ring-cleavage of the N-glycosidically bound GlcNAc (loss of 120 u from the peptide+GlcNAc ion) to distinctly identify the peptide and the peptide+GlcNAc ions. The two latter approaches were suggested in another workflow of glycopeptide fragment analysis (Hoffmann et al. 2018) and the characterization of bisecting GlcNAc has also recently been described (Dang et al. 2019).

A limitation for all LC-MS/MS approaches for global glycopeptide analysis is that, in general, monosaccharide isomerism as well as glycosidic linkage conformation and ring position cannot be completely resolved. For instance, a HexNAc ion could come from a terminal GalNAcα3 residue, as part of the HBGA A epitope or from GlcNAcβ3, as part of the type 1 (Galβ3GlcNAcβ3) or type 2 (Galβ4GlcNAcβ3) internal structures. An analysis of structural isomerism of monosaccharides and glycosidic linkages of each glycan at each attachment site of each glycoprotein would however demand an immense, presently unrealistic, effort of isolating individual glycoproteins of individual HIEs, digesting them into peptide fragments and separating the various glycopeptides in amounts sufficient for methylation analysis and NMR-spectroscopy. Glycomic analyses would be an alternative for structural characterization of released glycans but would also demand amounts not easily obtained from these HIEs and, more importantly, would require extensive purification of individual glycoproteins to obtain any information on the glycans at specific glycosites of the core proteins. Therefore, the isomers of precursor ions and annotated fragments presented in this glycoproteomic study are based on recent literature criteria for interpretations of MS/MS data but partly also building on existing knowledge of the biosynthesis of these glycan core structures and the corresponding HBGA epitopes.

For comparison of our glycoproteomics results with the literature data, we found only one publication, by Finne et al. from 1989 (Finne et al. 1989), presenting a detailed structural characterization of human small intestinal polyfucosylated glycoproteins. In this early publication glycopeptides from delipidated intestinal epithelial cells obtained from intestinal resections of proximal ileum of one blood group A Lewis positive secretor and of proximal jejunum of one blood group O Lewis negative secretor, were analyzed. Glycopeptides were obtained after extensive digestion with pronase, separated and characterized by gel filtration and lectin affinity chromatography steps, and then a complex subfraction of each intestinal preparation was analyzed by methylation analysis, mass spectrometry and ^1^H-NMR spectroscopy with a special focus on characterizing fucosylated HBGA structures. The general conclusion was that the identified polyfucosylated N-glycans preferentially contained tri- and tetra-antennary structures with a bisecting GlcNAc residue and an α1,6-fucosylation of the core GlcNAc residue. The HBGA epitopes were related to the blood groups of the two individuals and their structures were defined as both of the type 1 and the type 2 chains carrying one to three fucoses in extended GalGlcNAc chains. These data are in very good agreement with our MS-based glycoproteomic findings from which we can identify the ABO(H) and Lewis antigens but not strictly differentiate between the type 1 and the type 2 chain HBGA epitopes. The fact that we identified mainly bi- and tri-antennary structures while Finne et al. reported a dominance of tri- and tetra-antennary structures may be more related to differences in both preparation strategies and analytical tools rather than differences in the biological material. However, differences in glycosylation along the length of the small intestine (our HIEs were all established from jejunal biopsies), the disease state of the patients and the possible influences of variations in the colonizing microflora cannot be excluded. It is important to bear in mind that the HIEs are *ex vivo* models of the human gut and the glycosylation status may be dependent on the degree of cellular differentiation, cell surroundings and extracellular matrix but also of nervous, endocrine or microbiome influences (Nagao-Kitamoto et al. 2020). However, our study has clearly shown that the HIEs, established from intestinal stem cells, express several membrane glycoproteins carrying site-specific, naturally occurring N-glycans of both pauci- and oligomannose, hybrid- and complex-types, the latter most often with a high level of fucosylated HBGAs and an almost complete lack of sialylation.

Our approach for structurally defining the HBGA epitopes of individual HIEs has provided a strong but not absolute correlation to the known ABO, Lewis and secretor geno- and phenotypes decisive for effective GII.4 HuNoV infection (Table 1) (Haga et al. 2020). The ABO status does not by itself correlate with susceptibility to the virus (1J, J6 and J10 are all of blood group A; J2 is of blood group B; J4, J4FUT2 and J8 of blood group O). Additionally, Lewis status does not by itself correlate with viral susceptibility (J2, J4, J4FUT2, J6 and J8 are all Lewis positive whereas 1J and J10 are Lewis negative). However, the 1J, J2, J6 and J4FUT2 HIEs, all secretors, allow for infection whereas J4, J8 and J10, all non-secretors, do not, again pointing to the importance of the secretor status for susceptibility to GII.4 HuNoV infection.

The HBGA epitopes varied distinctly between the individual HIEs partly dependent on the summed effect of ABO, Lewis and secretor status irrespective of which of the four glycoproteins TSN8, CEAM5, SUIS or AMPN that we analyzed (Figs 3 and 4). The correlation of HBGA epitopes to the secretor status, *FUT2* coding for an α1,2-fucosyltransferase typically expressed in intestinal epithelia and having a preference for the type 1 chain precursor, argues for HBGA epitopes being mainly of the type 1 chain in J2, J6 and J4FUT2 HIEs, all susceptible to the GII.4 HuNoV infection. This conclusion about the type 1 chain is further supported by the correlation between the appearance of difucosylated HBGA epitopes found in secretor and Lewis positive J2, J6 and J4FUT2 HIEs and the strong expression of both the secretor *FUT2* α1,2-fucosyltransferase and the Lewis α1,3/4-fucosyltransferase, coded for by the *FUT3* gene, in J2 HIEs, for which we have RNA-Seq gene expression data (Supplementary Table S3 and Supplementary Fig. S1). However, the additional expression of α1,3-fucosyltransferases (*FUT4*, *FUT6*, *FUT10*, *FUT11*) in J2 HIE indicate the likely biosynthesis of both fucosylated type 1 and type 2 chain structures among the hybrid- and complex-type N-glycans of these HIE glycoproteins. Indeed, the transcriptomic analyses of J2 cultures also identified weak expression of the *FUT1* gene, coding for an α1,2-fucosyltransferase, typically found in erythroid progenitor cells and preferentially fucosylating type 2 chain precursors, which supports the additional presence of type 2 chain HBGA epitopes in at least some of these HIEs. For instance, the presence of difucosylated epitopes in 1J HIE being Lewis negative, and thus lacking the *FUT3* encoded α1,4-fucosyltransferase activity, indeed supports the presence of difucosylated type 2 chain structures in these cells. Interestingly, the J4 cells being non-secretors had originally both mono- and difucosylated structures but typically increased its content of difucosylated structures when transduced with a functional *FUT2* construct illustrating the dynamics in the biosynthesis of these HBGA epitopes.

Our results agree partly with the detailed characterization of HBGA epitopes of glycosphingolipids for these particular HIEs, that we recently described (Rimkute et al. 2020). However, for the glycosphingolipids the HBGA pattern was simpler since only type 1 and no type 2 chain structures were identified. As to the relevance of the HIEs as a model for studying intestinal epithelia, the glycolipid and glycoprotein data fit very well with the HBGA structures characterized from human small intestinal biopsies taken from single individuals with different HBGA status (Bjork et al. 1987; Finne et al. 1989; Breimer et al. 2012).

The very low levels of sialylated N-linked glycans of membrane glycoproteins of the HIEs is noteworthy since intestinal mucins are considered to have a very high content of both sialylated N- and O-linked glycans (Robbe et al. 2004; Nummela et al. 2021). However, very little is known about human jejunal tissues (Finne et al. 1989), the tissue from which the HIEs described here were established. Interestingly, the RNA-Seq data (Supplementary Table S3) give support for the presence of both α2,3-, α2,6- and α2,8-sialyltransferase activities with a dominance of expression of the *ST3GAL1*, *ST6GALNAC1* and *ST6GALNAC4* genes*.* If the mRNA expression levels does correlate with the protein expressions one may conclude that the conditions are there for sialylation to occur, especially for the biosynthesis of sialylated epitopes typical for mucin type O-glycosylations. However, in this project we did not study the mucins but rather the membrane bound N-glycoproteins of the HIEs and their post-translational modifications in the Golgi are probably different from that of the mucins. Another possible explanation may be the presence of neuraminidases. The RNA-Seq data also show that transcripts for NEU1, but not NEU 2-4, are expressed (Supplementary Table S3). However, none of the four neuraminidases identified in human tissues (NEU1-4) have been found at any higher amounts in small intestinal tissue but rather in muscle tissue, liver, brain, kidney, pancreas, adrenal glands or thymus where they operate optimally at different acidic pH from 3.2 to 6.5 and in different subcellular locations (Okun et al. 2023). Thus, apart from being a highly energy wasting procedure, including both biosynthesis and degradation, the lack of sialylated N-glycoproteins resulting from cellular activities of neuraminidases seems less likely than a consequence of high fucosyltransferase activities in the HIE cultures studied.

Released O-linked glycans of mucins of the human ileum, caecum and colon were recently studied by Robbe et al. (Robbe et al. 2004), and among the >100 complex oligosaccharides identified, the authors observed a gradient of more fucosylated structures in the ileum and of more acidic glycans, sialylated or sulfated, in the colon. A study of the N-linked glycans obtained from biopsies from terminal ileum and six locations of the human colon, corroborated this change in glycosylation pattern with fucosylated (H and Lewis b/y) glycans, often with bisecting GlcNAc structures dominating in the proximal parts and core fucosylated and sialylated structures dominating in the more distal (caudal) parts (Stavenhagen 2017). Such glycosylation gradients and their correlation to the intestinal microbiome was discussed in a recent review on intestinal membrane mucins (Pelaseyed and Hansson 2020).

Our study has provided the first global information on glycosite specific N-glycan structures of 93 glycoproteins from 7 HIE cultures established from 6 different individuals. The purpose of the study was not to define as many glycans or glycoproteins as possible but rather to define the major carriers of HBGA epitopes among membrane glycoproteins of human intestinal enteroids, which are either resistant or susceptible to HuNoV infection. Complex-type N-glycans carrying Fuc were found in 24 glycoproteins and 4 of those glycoproteins were characterized in detail giving complex patterns of site-specific glycosylations relating to the HBGA status of the individual HIEs. The fact that these glycoproteins are found in all HIEs analyzed, whether susceptible to HuNoV or not, implies that the putative protein receptor is expressed in HIEs regardless of virus susceptibility, but indeed requires correct fucosylations for infection to take place. The identification of these intestinal glycoproteins and their site-specific glycosylations, has given us a novel and detailed information on the complex pattern of glycosylation of human intestinal epithelial cells cultured *ex vivo*. Additionally, our study has provided a broad platform to advance our search for finding a functional receptor for HuNoV in intestinal target cells.

## Materials and methods

### HIEs

Establishment of HIE cells from patient tissues was originally described by Dr. Hans Clevers group (Sato et al. 2009; Sato et al. 2011; Sato and Clevers 2013), and maintenance, expansion and differentiation of cell cultures, resistant or susceptible for HuNoV replication, has already been reported (Ettayebi et al. 2016; Estes et al. 2019; Zou et al. 2019; Ettayebi et al. 2021). Cells of differentiated jejunal HIEs from six separate individuals (named 1J, J2, J4, J6, J8, J10) and from J4 HIEs transduced with the *FUT2* gene (Haga et al. 2020) were collected. Ethical approvals were obtained from the Institutional Review Board of Baylor College of Medicine and Affiliated Hospitals.

### Membrane protein extraction from HIEs

3D HIEs in Matrigel (Corning) were differentiated for five days prior to membrane-associated protein extraction with the Mem-PER Plus Membrane Protein Extraction Kit (#89842, Thermo Scientific) performed according to the manufacturer’s protocol. Generally, at least six wells of 3D HIEs (with ~3×10^5^ cells per well) in a 24-well plate were pooled together for one extraction. Matrigel was removed by washing the HIEs with 1X cold PBS and centrifugation for five min at 300 × g at 4 °C, and then the pelleted HIEs were suspended in the wash solution as the first step in the kit protocol. Next, 1X protease inhibitor (#539134, Calbiochem) was added to the permeabilization and solubilization buffers. The final solubilized supernatants containing the membrane-associated proteins were stored at −80 °C until processing for glycoproteomics analysis. The protein concentration was determined by OD values using the Nanodrop technology (Thermo Scientific).

### LC-MS/MS setup

The preparative method used has been previously described (Wohlgemuth et al. 2009; Mysling et al. 2010; Scott et al. 2011) but was slightly modified. Briefly, cell membrane lysates of individual HIEs were subjected to trypsin digestion and hydrophilic interaction liquid chromatography (HILIC) to enrich for tryptic glycopeptides.

Aliquots containing about 50 μg of each sample were digested with trypsin using the filter-aided sample preparation (FASP) method (Wisniewski et al. 2009). Protein extract samples containing 4% sodium dodecylsulfate (SDS), were reduced with 100 mM dithiothreitol at 60 °C for 30 min, transferred onto 30 kDa MWCO Nanosep centrifugal filters (Pall Life Sciences, Ann Arbor, USA), washed with 8 M urea solution and alkylated with 10 mM methyl methane-thiosulfonate in 50 mM triethylammonium bicarbonate (TEAB) buffer with 1% sodium deoxycholate. Digestion was performed in 50 mM TEAB, 1% sodium deoxycholate at 37 °C in two step incubations (trypsin:protein ratio 1:100) of Pierce MS-grade trypsin (Thermo Scientific) for 3 h, then additional trypsin was added and the digestion was performed overnight. The peptides were collected by centrifugation, dried and dissolved in 80% acetonitrile (ACN), 0.1% trifluoroacetic acid. In-house packed ZIC-HILIC SPE, 200 Å columns (Merck Sequant) were equilibrated with the dissolution solvent, sample loaded, washed in dissolution solvent and finally the glycopeptides were eluted once with 5% ACN 1% formic acid and a second elution with 1% formic acid.

The glycopeptides were analyzed with an Orbitrap Fusion Tribrid mass spectrometer interfaced with Easy-nLC 1200 nanoflow liquid chromatography system (Thermo Fisher Scientific). Peptides were trapped on the Acclaim Pepmap 100 C18 trap column (100 μm × 2 cm, particle size 5 μm, Thermo Fischer Scientific) and separated on an in-house packed C18 analytical column (75 μm × 30 cm, particle size 3 μm) using a gradient from 7% to 35% B during 70 or 75 min, from 35% to 100% B in 5 or 10 min and then 100% B for 10 min. Solvent A was 0.2% formic acid and solvent B was 80% ACN, 0.2% formic acid. Precursor ion mass spectra were recorded between *m/z* 600–2,000, charge states 2–7 at 120,000 resolution, the most intense precursor ions were selected (“top speed” setting with a duty cycle of 3 s), fragmented using HCD at collision energy setting of 35% with stepped collision energy of ±15%; or alternatively consecutively at HCD 30% and 40% on the same precursor ion. The MS/MS spectra were detected in the Orbitrap with the maximum injection time of 60 ms or 118 ms and the isolation window of 2.5 or 5 *m/z* units.

### Glycoproteomics analysis

MS/MS database searches were done with the Byonic software (Protein metrics) within Proteome discoverer 2.4 (Thermo Scientific) using an in-house list of 243 N-glycan modifications (Supplementary Table S5). Glycan compositions included oligomannose, hybrid- and complex-type N-glycans carrying 1–6 fucose (Fuc) and 0–3 additional HexNAc and/or Hex residues to account for possible A, B, H and Lewis structures. A second Byonic analysis was conducted with 96 N-glycan compositions including 1–4 Neu5Ac residues. Additional allowed modifications were methionine oxidation and static methylthio-cysteine. Trypsin digested peptides were searched using the human sequences of the Swiss-Prot database (May 2022, 20,307 proteins), at a 5 ppm mass limit of the precursor ions and a 20 ppm limit of the MS^2^ ions. A false discovery rate (FDR) of 2% (individual spectral matches) and 1% (peptide groups) were set; and an additional Byonic score cut-off of >200 and a PEP-2D score < 0.001, in at least one of the analyzed samples, was used for glycopeptide matches. All HBGA and related complex-type glycopeptide identities were manually verified to 1) contain the presence of the correct peptide+HexNAc ion; 2) secure identification of Fuc (dHex; deoxyhexose) residues bound to the glycan core (presence of peptide+HexNAc+dHex ion) and/or to the glycan antennae (presence of *m/z* 512.20 ion) of fucosylated glycopeptides and 3) present, for glycopeptides sharing the same peptide sequence, identification of at least four b- and/or y-ions for the best scoring glycopeptide hit (Parker et al. 2013). Further, HBGA and related complex-type N-glycopeptide identities were included also for PEP-2D scores >0.001 if they passed the manually set criteria and if alternative glycoforms of the same peptide with a PEP-2D score < 0.001 were identified. For global glycopeptide structure profiles and manual identification of additional glycoforms, extracted ion chromatograms were prepared by tracing selected saccharide oxonium ions and selected peptide+HexNAc ions at the MS^2^ level with the Xcalibur software (Thermo Scientific). Traced oxonium ions included *m/z* 163.06 (at >20% relative abundance of the oxonium ions for oligomannose structures), *m/z* 204.09 (general for HexNAc containing glycans), *m/z* 274.09 (specific for sialic acid (Neu5Ac)), and HBGA specific ions at *m/z* 512.20; 658.26; 674.25; 820.31; 861.33; 877.33; 1023.39; and 1039.38 (see Table 3). The relative quantifications of the major HBGAs (and related) N-glycoforms were done by concurrent tracing of the relative abundances of up to three of the most abundant precursor ions, sharing the same peptide, across their retention time spans.

### RNA-Seq analysis

The RNA-Seq data were from a previously published dataset (Lin et al. 2020). Briefly, RNA-Seq analysis performed on uninfected (mock treated) 5-day differentiated monolayer HIE cultures. A cDNA library was prepared from extracted total cellular RNAs that were first depleted of cytoplasmic and mitochondrial ribosomal RNA. Paired end sequencing (2 × 100 bp) was performed on an Illumina HiSeq 2,500 for a depth of 30 million paired end reads (a total of 60 million reads) per RNA sample. The fastq files generated during sequencing were quality trimmed using Trim Galore. Trimmed reads were then aligned to GRCh38 using Hisat2 and then a count matrix was generated from the aligned reads using featureCounts. The RNA-Seq data files and raw count matrix files were deposited in the Gene Expression Omnibus (GSE150918).

## Supplementary Material

HIE_glp_suppl_FINAL_Revision_AcceptedChanges_cwae029

HIE_glp_Revised_Table_S1_FINAL_cwae029

HIE_glp_Revised_Table_S5_FINAL_cwae029

HIE_glp_Revised_Table_S6_FINAL_cwae029

## Data Availability

The RNA-seq data files and raw count matrix files were deposited in the Gene Expression Omnibus (GSE150918). The MS data and Byonic viewer files have been deposited to the ProteomeXchange Consortium via the PRIDE partner repository with the dataset identifier PXD046494. MS files, scan numbers and additional information for all the presented HBGA glycopeptides are provided in the Supplementary Table S6.
