# Supplementary material for: N-glycoproteomic analyses of human intestinal enteroids, varying in histo-blood group geno- and phenotypes, reveal a wide repertoire of fucosylated glycoproteins
Source: Glycobiology. 2024 Apr 9;34(6):cwae029. doi: 10.1093/glycob/cwae029 (PMC11041853; doi:10.1093/glycob/cwae029)
Supplement: HIE_glp_suppl_FINAL_Revision_AcceptedChanges_cwae029 [file hie_glp_suppl_final_revision_acceptedchanges_cwae029.pdf]

N-glycoproteomic analyses of human intestinal enteroids, varying in histo-blood group geno- and phenotypes, reveal a wide repertoire of fucosylated glycoproteins

Jonas Nilsson[1,2,3], Inga Rimkuté[1,4], Carina Sihlbom[3], Victoria R Tenge[5], Shih-Ching Lin[5*], Robert L Atmar[5, 6], Mary K Estes[5,6,§] and Göran Larson[1,2, §]

[1]Department of Laboratory Medicine, Institute of Biomedicine, University of Gothenburg, Sweden
[2]Laboratory of Clinical Chemistry, Sahlgrenska University Hospital, Gothenburg, Sweden
[3]Proteomics Core Facilities, Sahlgrenska academy, University of Gothenburg, Sweden
[4]Department of Microbiology and Immunology, Institute of Biomedicine, University of Gothenburg, Sweden
[5]Department of Molecular Virology, Baylor College School of Medicine, Houston, TX, USA
[6]Department of Medicine, Baylor College of Medicine, Houston, TX, USA

[*] Present address: Department of Medicine, Washington University in St. Louis, St. Louis, MO, USA

[§]Communicating authors: goran.larson@clinchem.gu.se; mestes@bcm.edu

Supplementary material, Figures

- **Figure S1**. Biosynthesis of type 1 and type 2 chain HBGA antigens.
- **Figure S2**. Examples of hybrid and complex glycoforms of tetraspanin-8 (TSN8) from the J2 HIE.
- **Figure S3.** Examples of MS^2 spectra of tetraspanin-8 (TSN8) *N*-glycopeptides from J6, J8, and J10 HIEs.
- **Figure S4.** Extracted ion chromatograms (XICs) of critical oxonium ions.
- **Figure S5.** Extracted ion chromatograms (XICs) of oxonium ions for histo-blood group B and A epitopes.
- **Figure S6.** Examples of MS^2 spectra of carcinoembryonic antigen-related cell adhesion molecule 5 (CEAM5) glycopeptides containing the Asn-204/560 glycosite from J2, J6, J8, and J10 HIEs.
- **Figure S7.** Examples of MS^2 spectra of sucrase/isomaltase (SUIS) glycopeptides from J2, J6, J8 and J10 HIEs.
- **Figure S8.** Examples of MS^2 spectra of aminopeptidase-N (AMPN) glycopeptides J2, J6, J8, and J10 HIEs.
- **Figure S9.** Examples of MS^2 spectra of aminopeptidase-N (AMPN) glycopeptides from J2, J6, J8, and J10 HIEs.
- **Figure S10.** Examples of MS^2 spectra of tetraspanin-8 (TSN8) *N*-glycopeptides from 1J, J4, and J4FUT2 HIEs.
- **Figure S11.** Examples of MS^2 spectra of carcinoembryonic antigen-related cell adhesion molecule 5 (CEAM5) glycopeptides containing the Asn-204/560 glycosite for 1J, J4, and J4FUT2 HIEs.
- **Figure S12.** Examples of MS^2 spectra of sucrase/isomaltase (SUIS) glycopeptides from J4, and J4FUT2 HIEs.

- **Figure S13.** Examples of MS^2 spectra of aminopeptidase-N (AMPN) glycopeptides from 1J, J4 and J4FUT2 HIEs.
- **Figure S14.** MS^2 identification of sialylated *N*-glycopeptides originating from Matrigel glycoproteins in a J2 HIE preparation.
- **Figure S15.** XIC MS^2 assay to investigate the presence of Neu5Ac glycoforms among the TSN8 glycopeptides of the J2 and J6 HIE preparations.

Supplementary material, Tables

- **Table S1.** Byonic MS data file (excel document submitted separately).

- **Table S2.** Protein characteristics of 23 of 24 glycoproteins carrying fucosylated N-glycans identified in the HIE cultures.
- **Table S3.** Expression of fucosyltransferase, sialidase (neuraminidase) and sialyltransferase mRNAs in J2 HIEs.
- **Table S4.** Sialic acid Neu5Ac and Neu5Gc N-glycopeptide identities from HIE preparations.

- **Table S5.** N-glycan composition database (excel document submitted separately).
- **Table S6**. MS data file sources for annotated figures and data availability, Byonic MS data file including spectral matches information, and Neu5Ac Byonic analysis (excel document submitted separately).

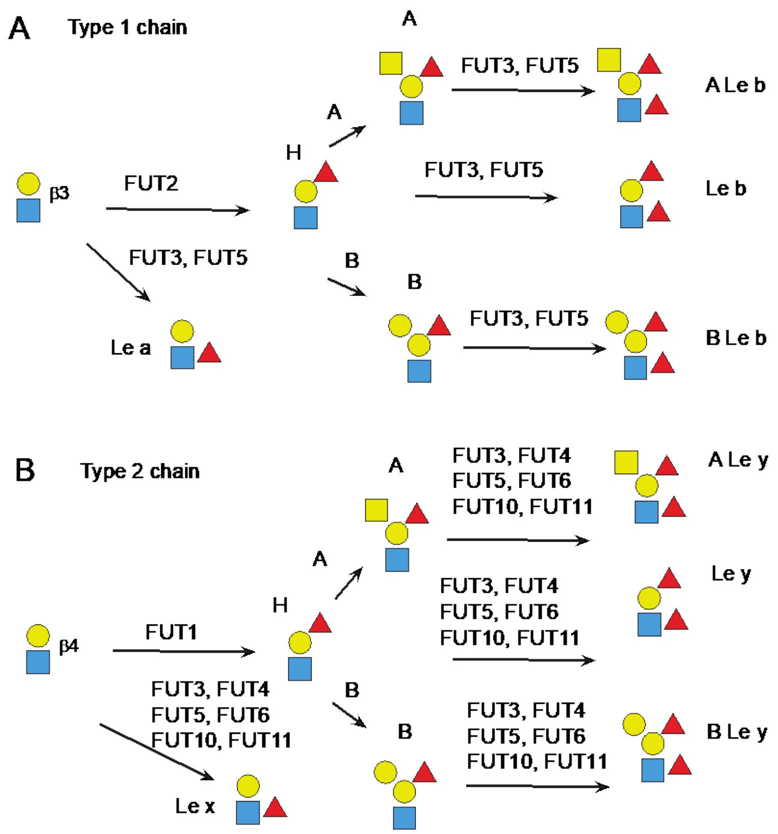

Figure S1. **Biosynthesis of type 1 and type 2 chain HBGA antigens.** The biosynthesis starts with either a type 1 chain Galβ1,3GlcNAc- (lactoseries) or a type 2 chain Galβ1,4GlcNAc- (neolactoseries) precursor structure that is fucosylated either at the terminal Gal to give the H type 1 or H type 2 epitopes, catalyzed by the α1,2-fucosyltransferases coded for by the *FUT2* and *FUT1* genes respectively, or at the subterminal GlcNAc to give the Lewis a and Lewis x antigens catalyzed by one of two α1,3/1,4-fucosyltransferases coded for by the *FUT3* Lewis or *FUT5* genes or by any one of the α1,3-fucosyltransferases coded for by the *FUT4*, *FUT6*, *FUT10* or *FUT11* genes, respectively. The H antigens produced may then similarly be modified by either the α1,3-N-acetylgalactosaminyltransferase coded for by the *ABO* gene to give the A antigen in A individuals or by the α1,3-galactosyltransferase coded for by the *ABO* gene to give the B antigen in B individuals. Alternatively, the H antigens can become fucosylated at the subterminal GlcNAc to yield the Lewis b and Lewis y antigens, respectively, catalyzed by the α1,3/4- or α1,3-fucosyltransferases mentioned above. For blood group O individuals the *ABO* gene is non-functional and thus extension to the A or B antigen is not possible and consequently fucosylation to the Le^b and Le^y are the only modifications allowed for the H antigens. Finally, the A and B antigens may also be modified with the same α1,3/4- or α1,3-fucosyltransferases to give the type 1 chain ALe^b or BLe^b or the type 2 chain ALe^y or BLe^y antigens, respectively. The α1,2-fucosyltransferase coded for by the *FUT2* gene is mainly expressed in epithelia and operates preferentially on the type 1 chain and is a prerequisite for the biosynthesis of ABO antigens in saliva and on mucosal surfaces, typically active in “secretors” (secretor positives) but not in “non-secretors” (secretor negatives). Therefore, the *FUT2* gene is often called the secretor gene.

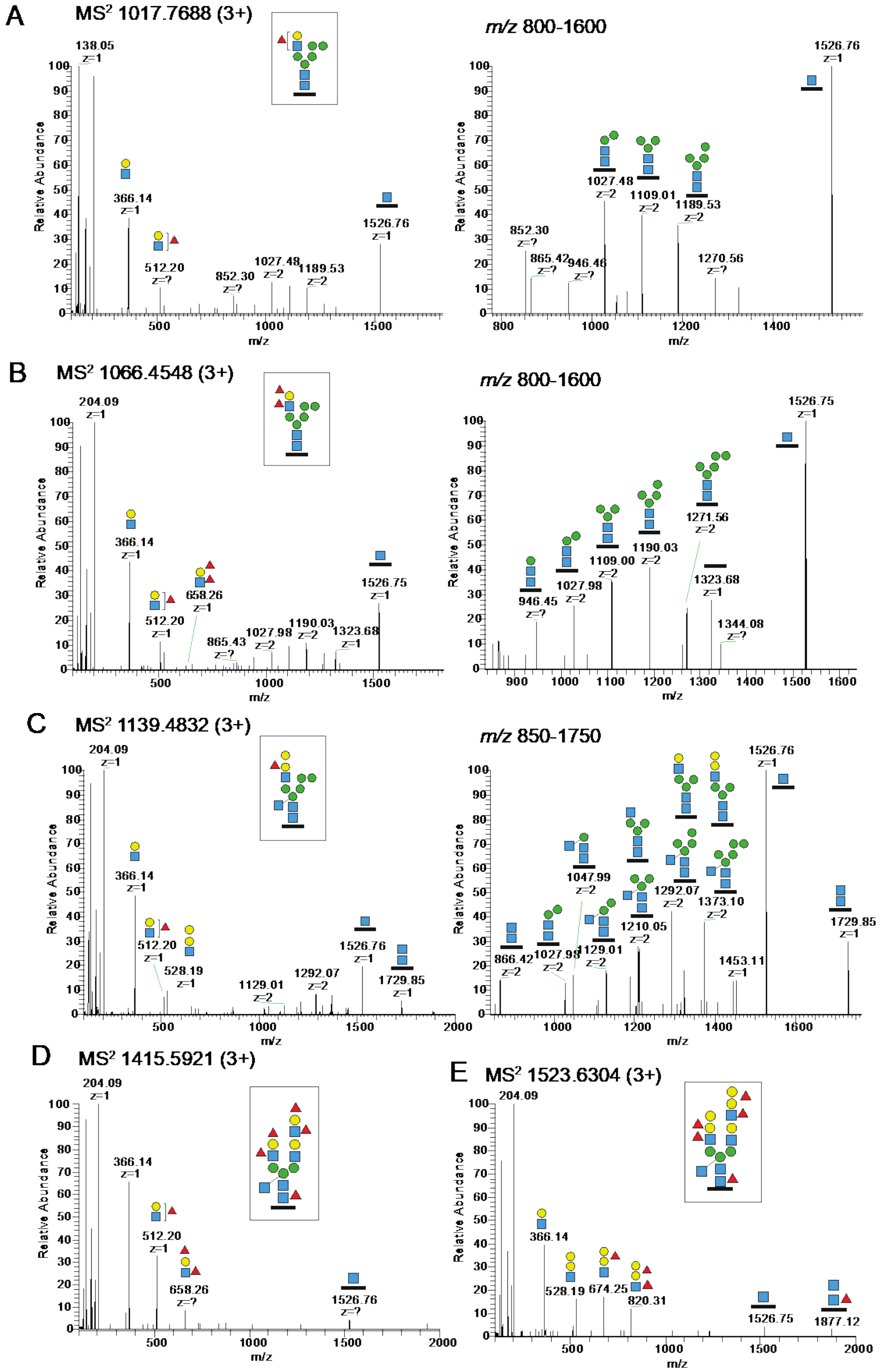

Figure S2. Examples of hybrid and complex glycoforms of tetraspanin-8 (TSN8) from the J2 HIE. The bisecting GlcNAc identity is demonstrated in **C**, right expanded spectrum (*m/z* 1047.99).

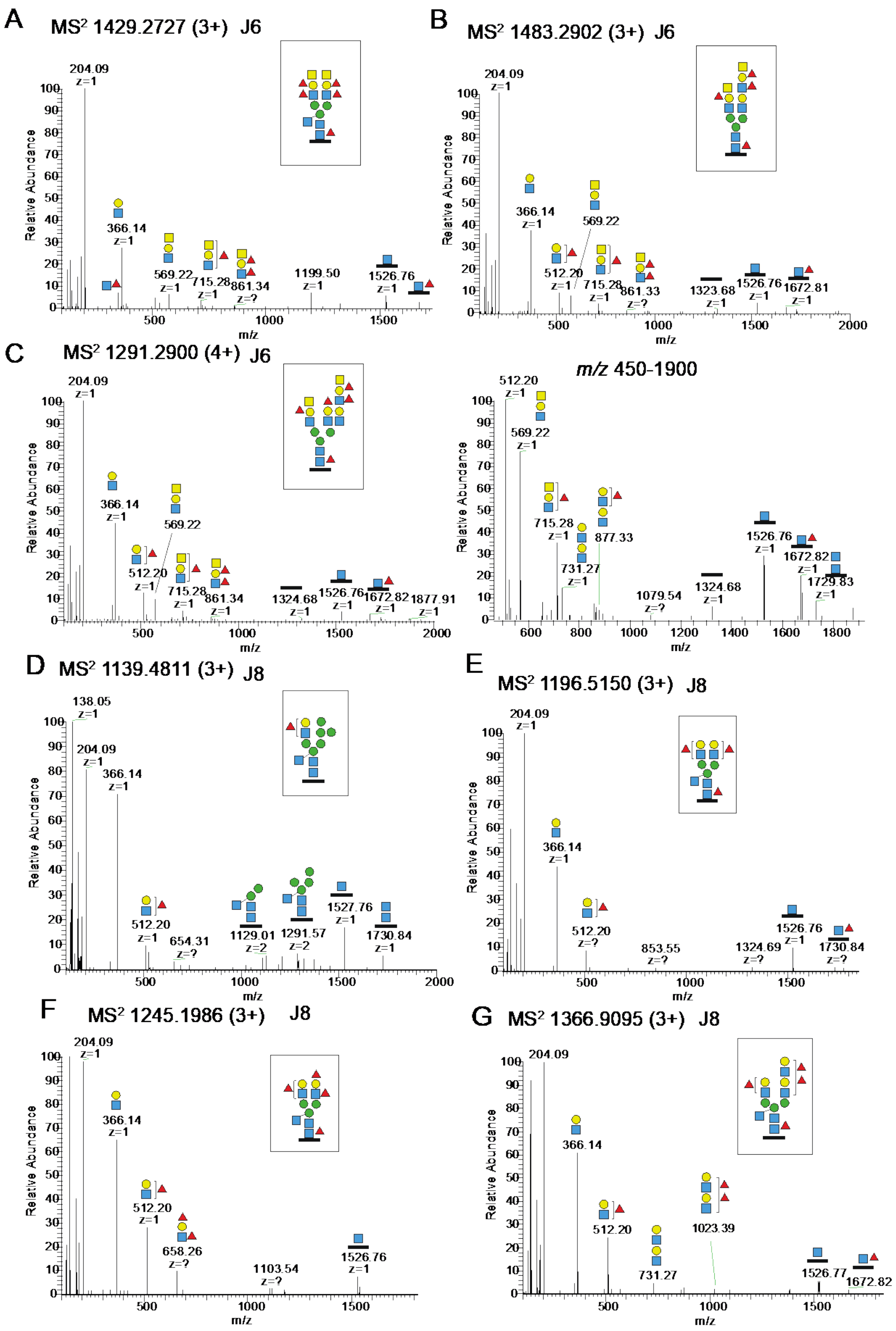

Figure S3. **Examples of MS² spectra of tetraspanin-8 (TSN8) *N*-glycopeptides** from (**A-C**) J6; (**D-G**) J8; and (**H-I**) J10 HIEs.

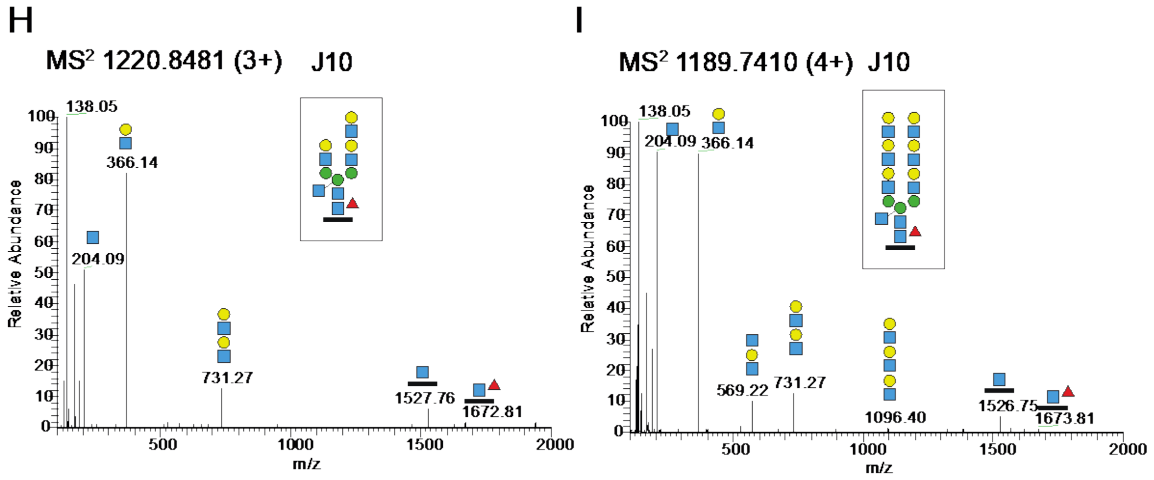

Figure S3 continued. Examples of MS^2 spectra of tetraspanin-8 (TSN8) *N*-glycopeptides from (**A-C**) J6; (**D-G**) J8; and (**H-I**) J10 HIEs.

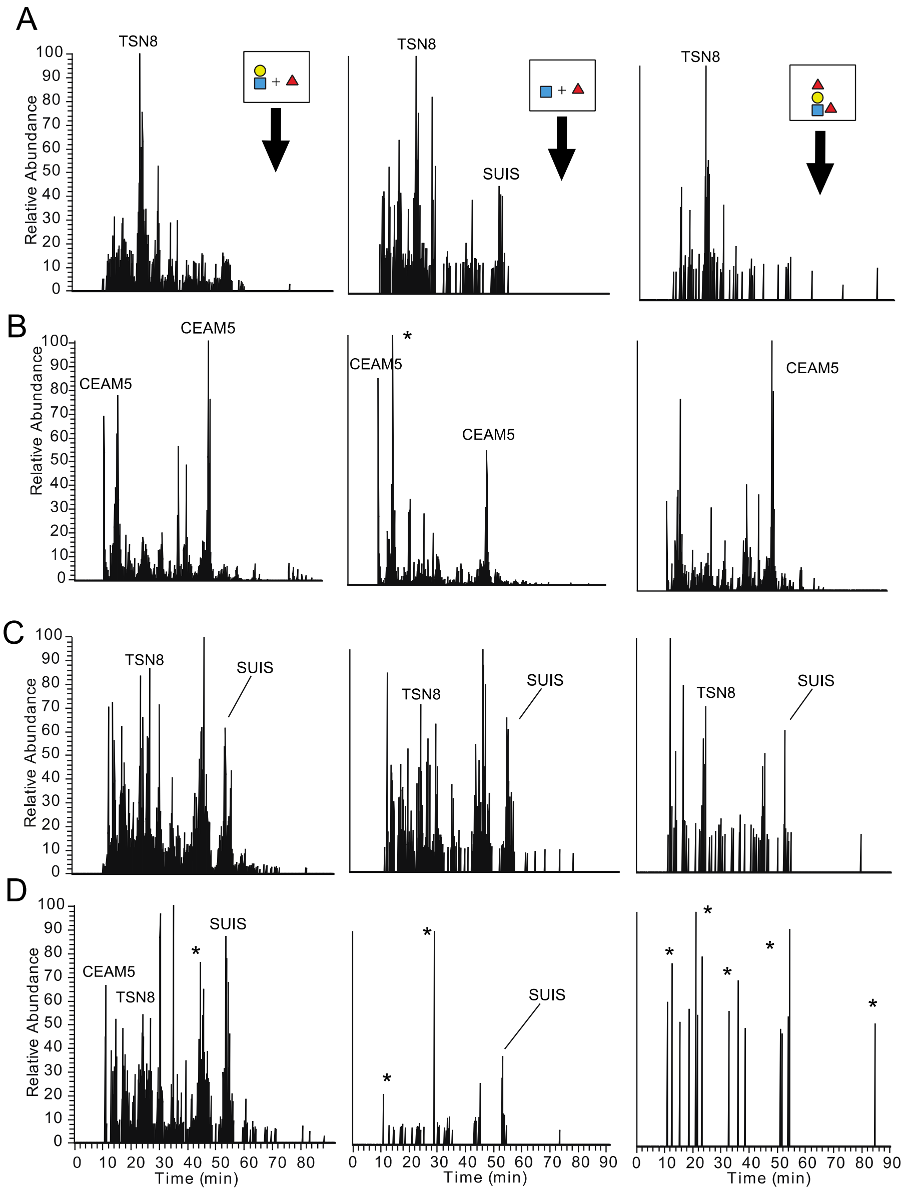

Figure S4. Extracted ion chromatograms (XICs) of diagnostic oxonium ions. XICs for the (**A**) J2; (**B**) J6; (**C**) J8; and (**D**) J10 HIEs. The composition of each oxonium ion is shown in (A), left to right, HexHexNAcdHex ion at *m/z* 512.20, HexNAcdHex ion at *m/z* 350.14, and HexHexNAc(dHex)$_2$ ion at *m/z* 658.26. Ions arising from prominent glycopeptides are indicated by their corresponding protein names (TSN8, CEAM5, SUIS). * Unrelated ion peaks.

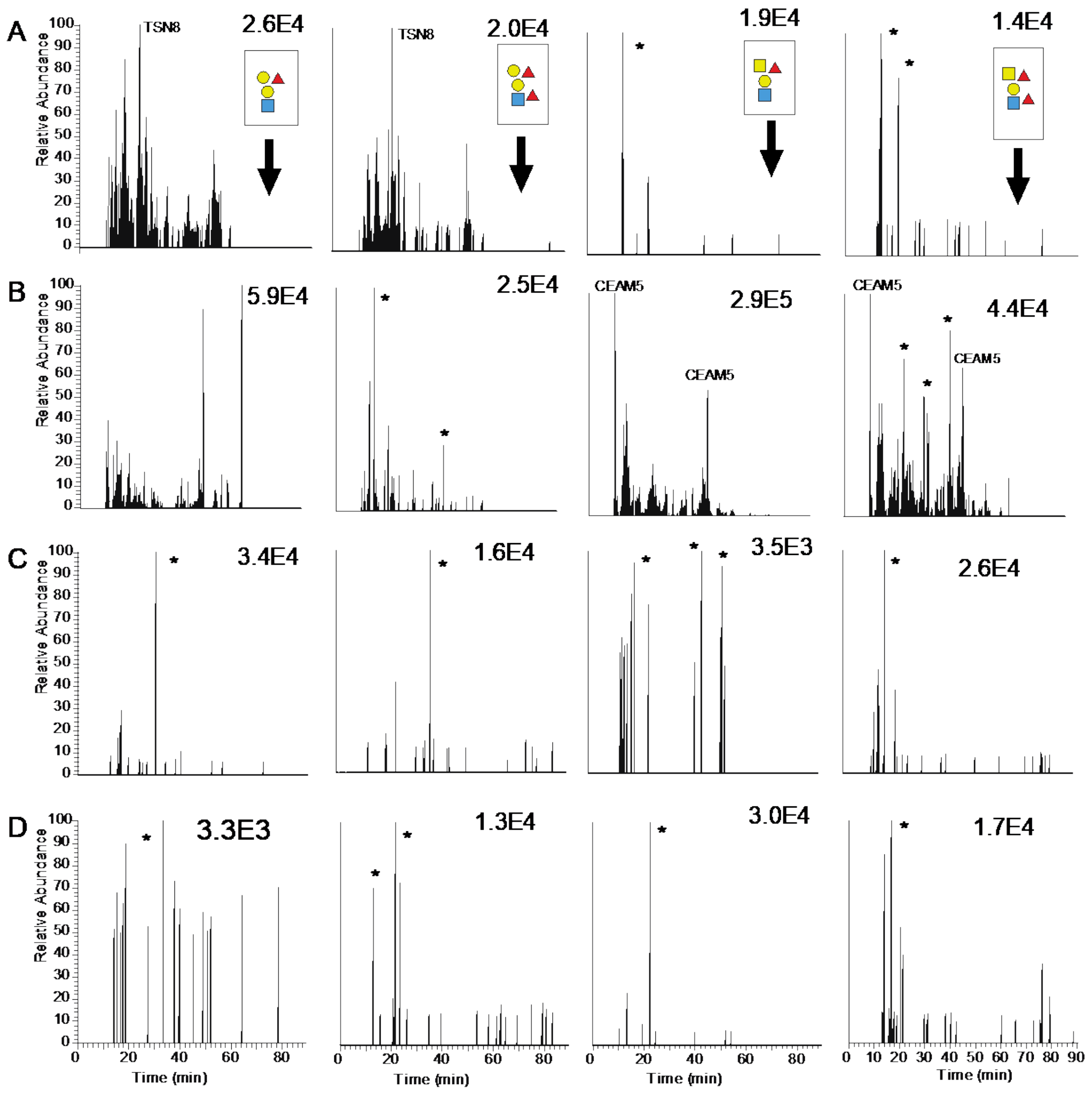

Figure S5. Extracted ion chromatograms (XICs) of diagnostic oxonium ions for histo-blood group B and A epitopes. XICs for the (**A**) J2; (**B**) J6; (**C**) J8; and (**D**) J10 HIEs. The composition of each oxonium ion is shown as inserts in (A) and corresponds to, from left to right, $Hex_2HexNAcdHex$ ion at *m/z* 674.25, $Hex_2HexNAcdHex_2$ ion at *m/z* 820.31, $HexHexNAc_2dHex$ ion at *m/z* 715.28, and $HexHexNAc_2dHex_2$ ion at *m/z* 861.33 * Unrelated ion peaks. The intensity (number of counts) of the largest ion peak is presented for each XIC. Ions arising from prominent glycopeptides are indicated by their corresponding protein names (TSN8, CEAM5).

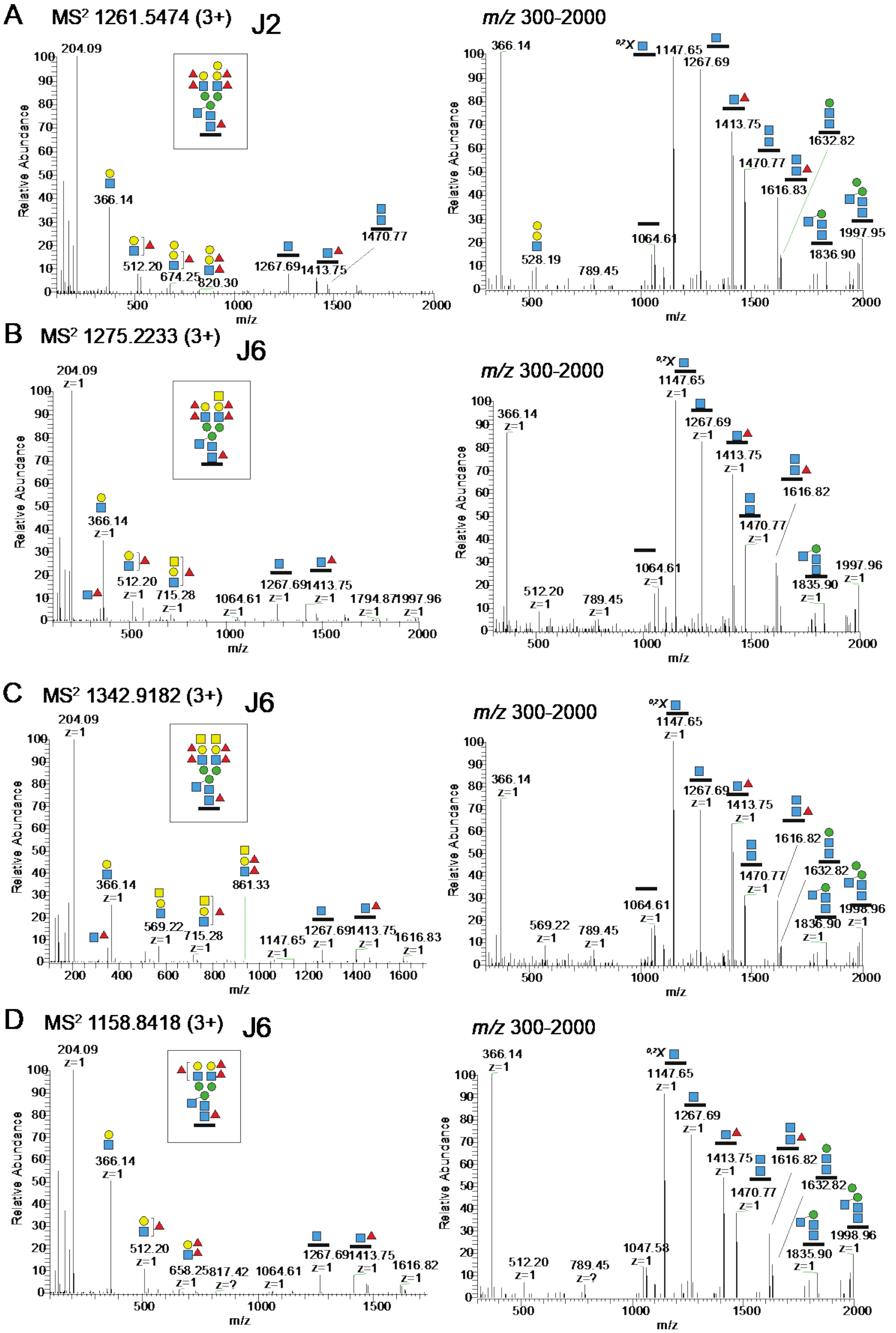

Figure S6. Examples of MS[2] spectra of carcinoembryonic antigen-related cell adhesion molecule 5 (CEAM5) glycopeptides containing the Asn-204/560 glycosite from (**A**) J2; (**B-D**) J6; (**E-F**) J8; and (**G-H**) J10 HIEs.

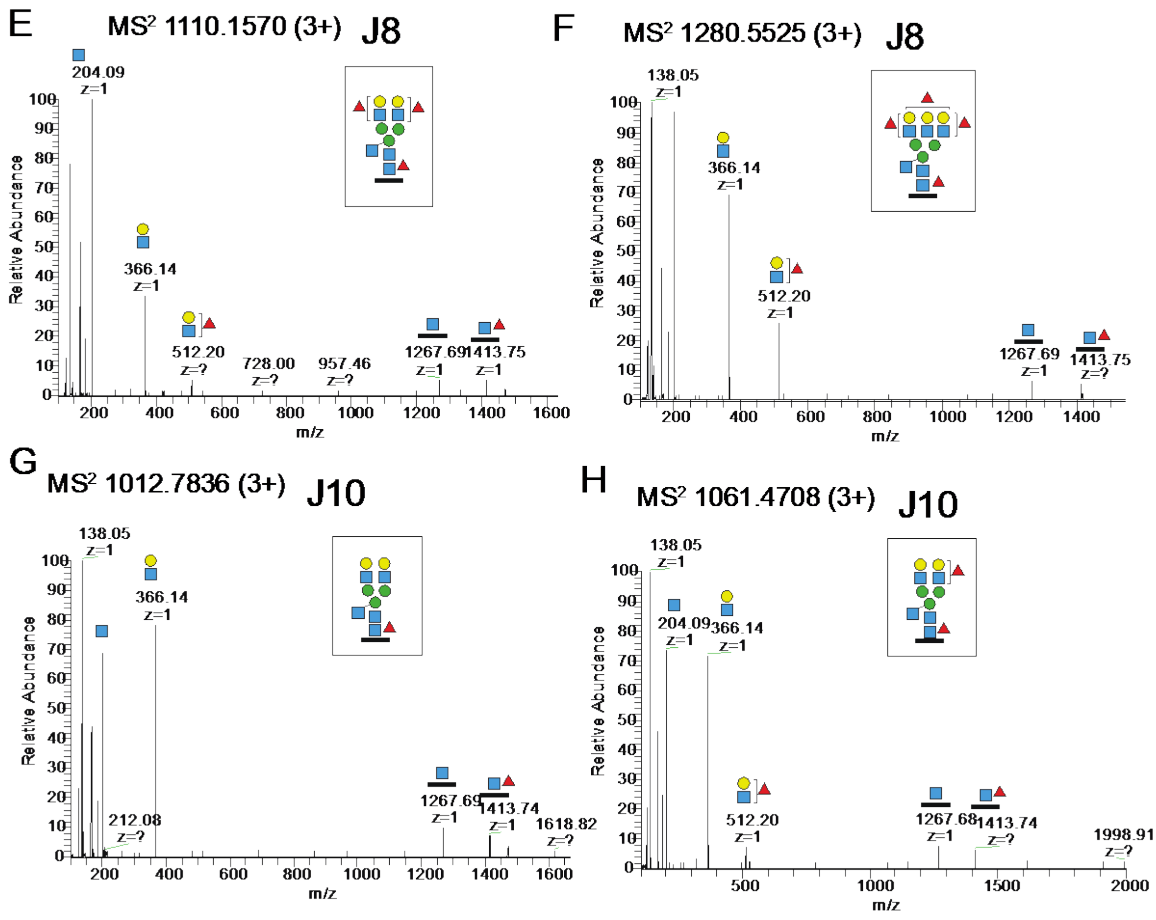

Figure S6 continued. Examples of MS^2 spectra of carcinoembryonic antigen-related cell adhesion molecule 5 (CEAM5) glycopeptides containing the Asn-204/560 glycosite from (**A**) J2; (**B-D**) J6; (**E-F**) J8; and (**G-H**) J10 HIEs. The presence of bisecting GlcNAc is demonstrated in A-D (right spectra, *m/z* 1835.90 / *m/z* 1836.90), which were acquired with a NCE of 40% whereas the left spectra were acquired with a NCE of 30%.

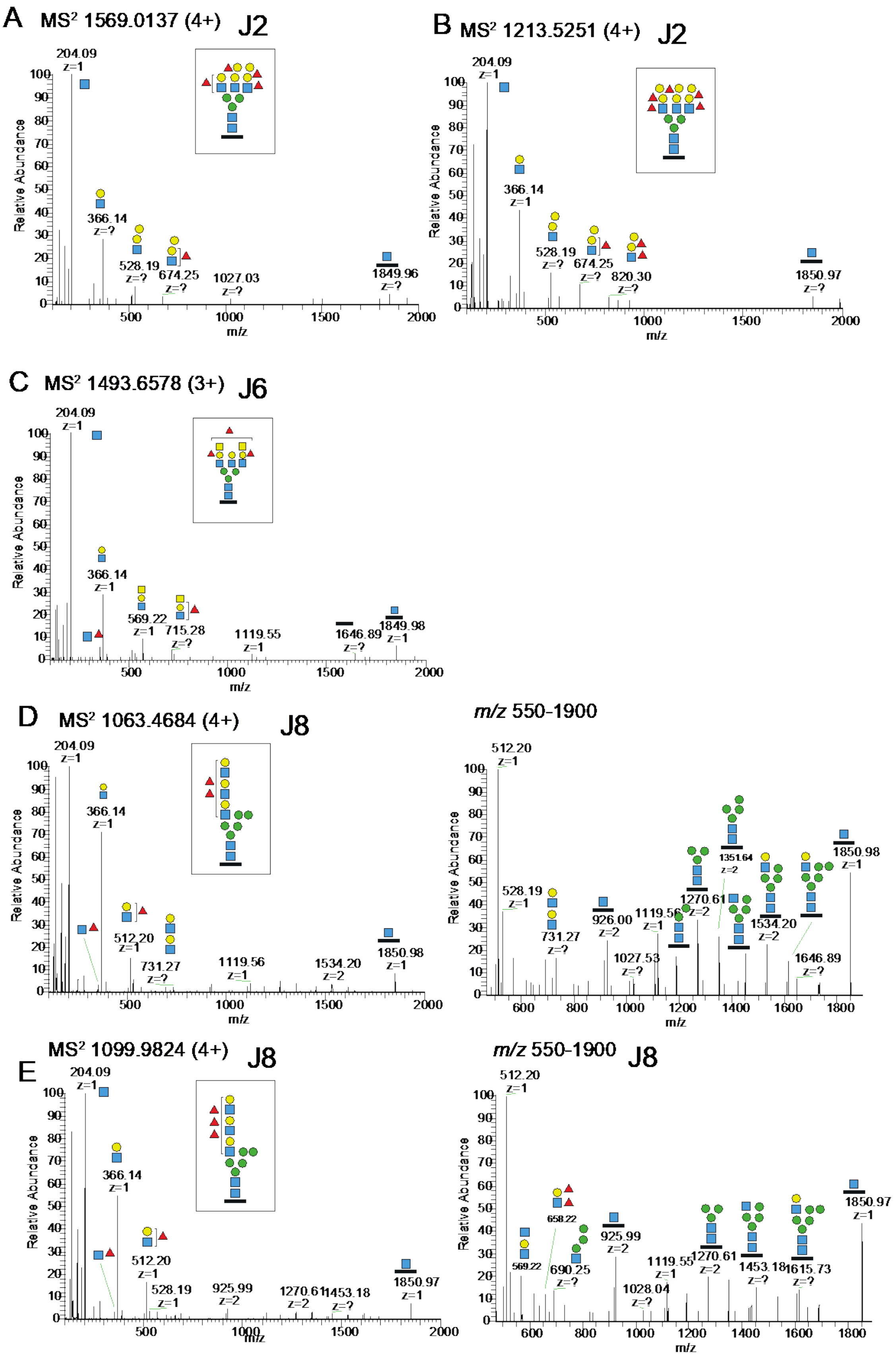

Figure S7. Examples of MS² spectra of sucrase/isomaltase (SUIS) glycopeptides from (**A-B**) J2, (**C**) J6, (**D-E**) J8 and (**F-G**) J10 HIEs.

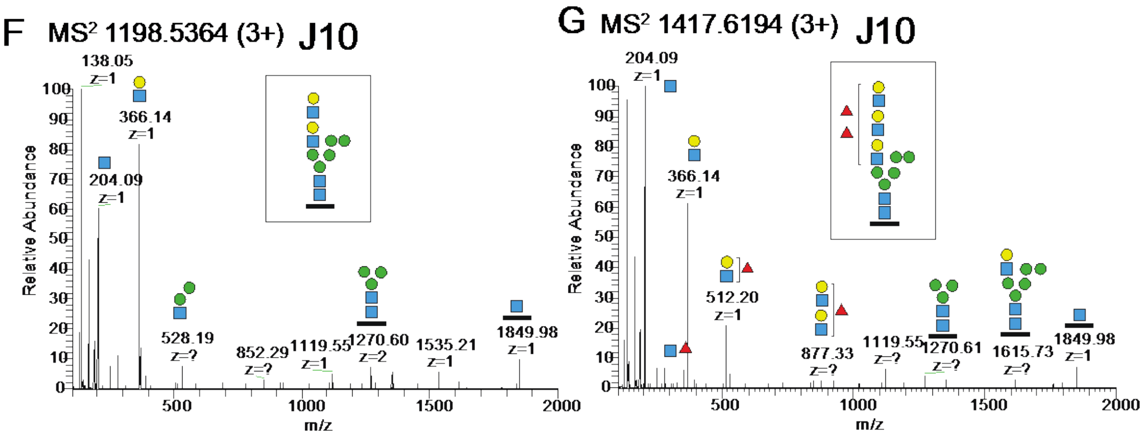

Figure S7 continued. Examples of MS^2 spectra of sucrase/isomaltase (SUIS) glycopeptides from (**A-B**) J2, (**C**) J6, (**D-E**) J8 and (**F-G**) J10 HIEs. The peptide sequence is YIIILDPAISGNETK including the Asn-1303 glycosylation site.

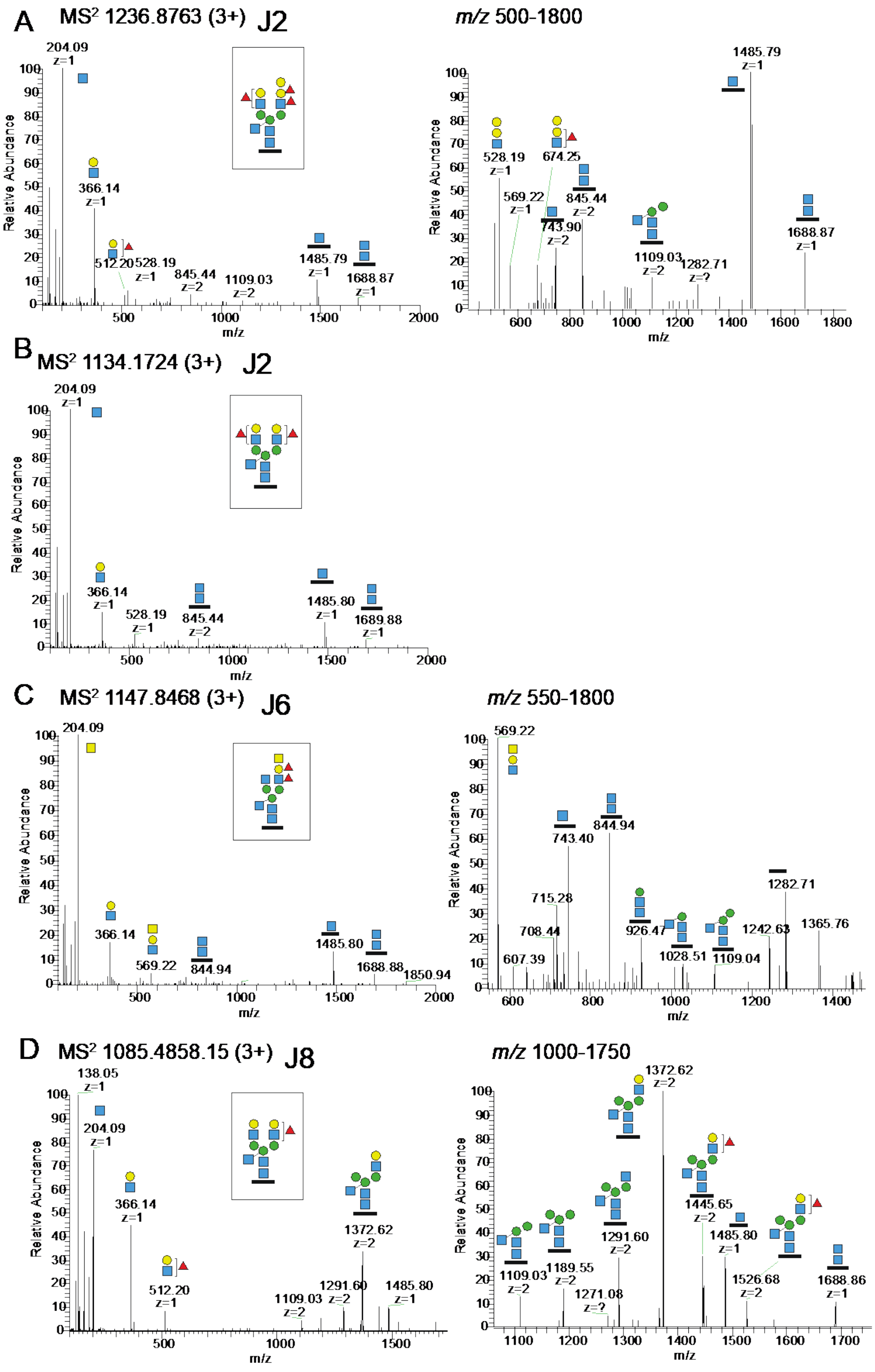

Figure S8. **Examples of MS^2 spectra of aminopeptidase-N (AMPN) glycopeptides** from (**A-B**) J2, (**C**) J6, (**D**) J8 and (**E-G**) J10 HIEs.

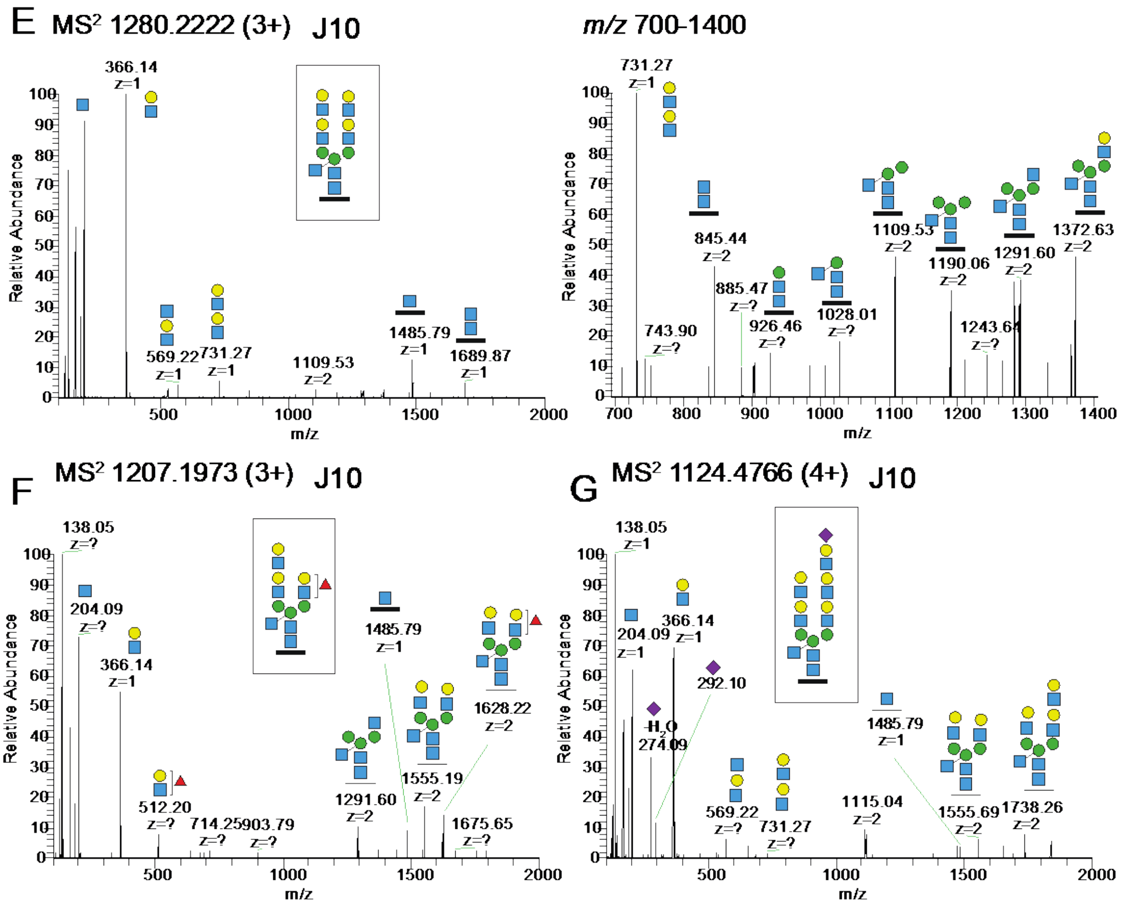

Figure S8 continued. Examples of MS^2 spectra of aminopeptidase-N (AMPN) glycopeptides from (**A-B**) J2, (**C**) J6, (**D**) J8 and (**E-G**) J10 HIEs. The peptide sequence is AEFNITLIHPK including the Asn-234 glycosylation site.

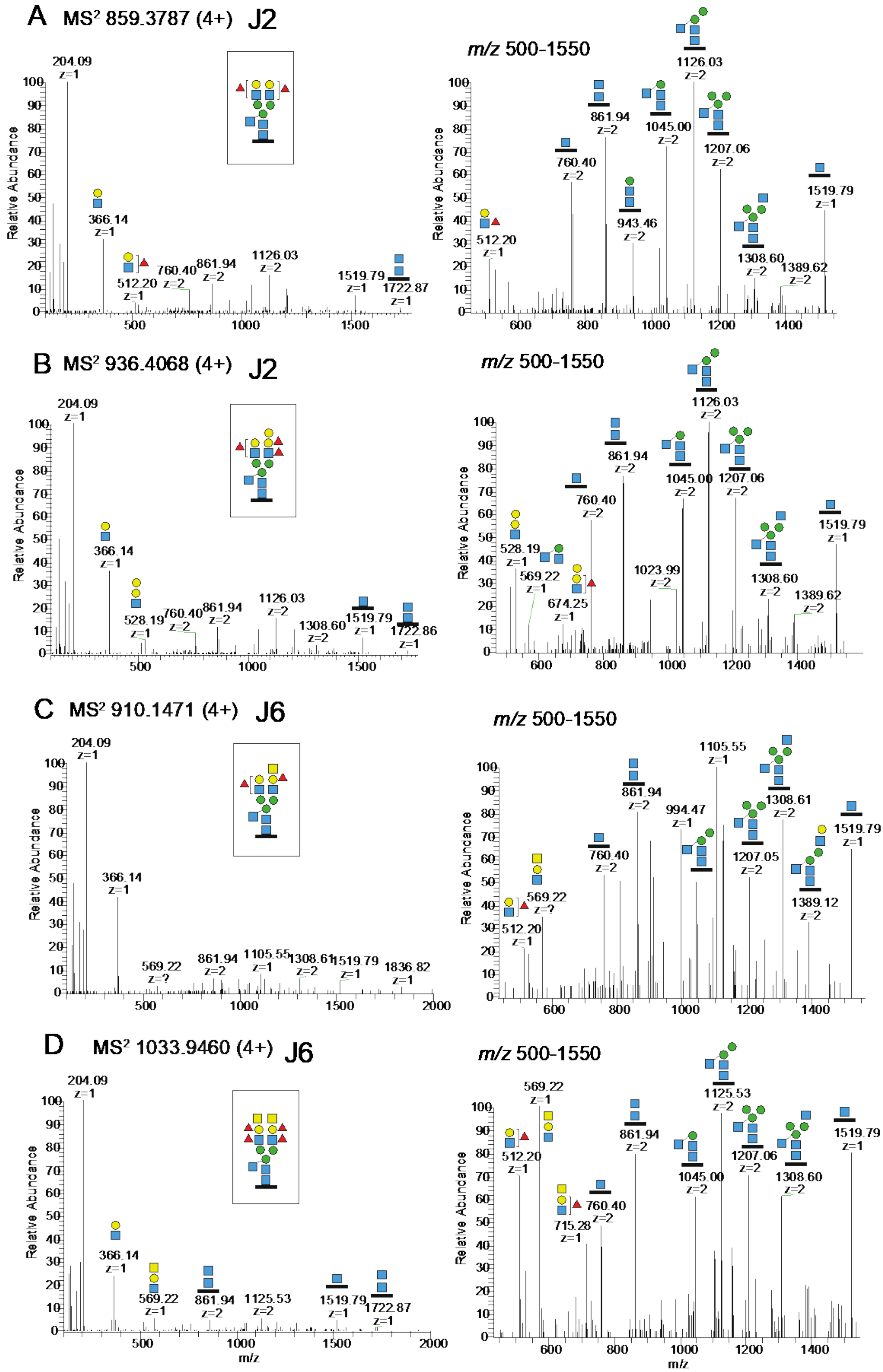

Figure S9. **Examples of MS² spectra of aminopeptidase-N (AMPN) glycopeptides** from (A-B) J2, (C-D) J6, (E-F) J8 and (G-H) J10 HIEs.

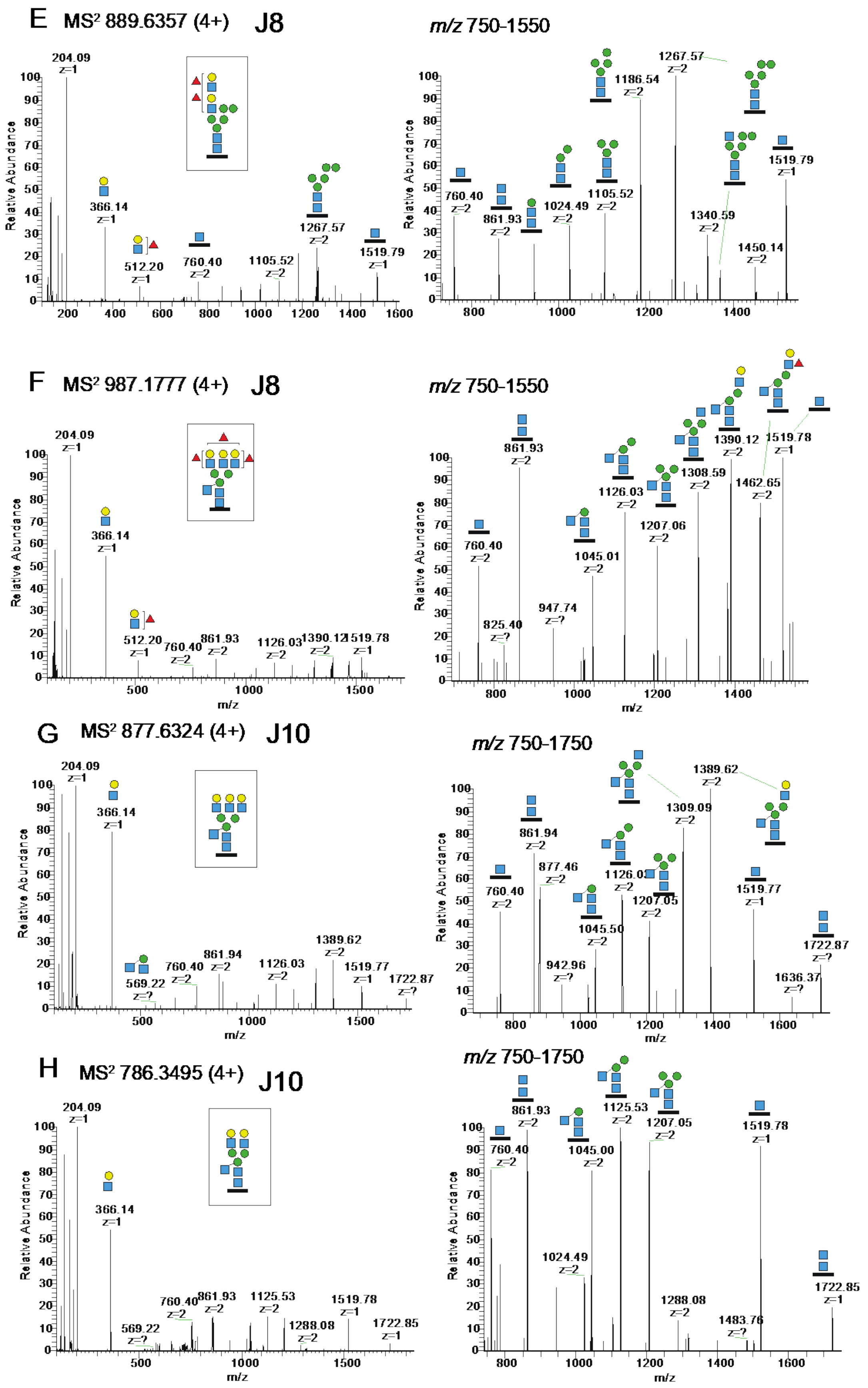

Figure S9 continued. **Examples of MS^2 spectra of aminopeptidase-N (AMPN) glycopeptides** from (A-B) J2, (C-D) J6, (E-F) J8 and (G-H) J10 HIEs. The peptide sequence is KLNYTLSQGHR including the Asn-128 glycosylation site.

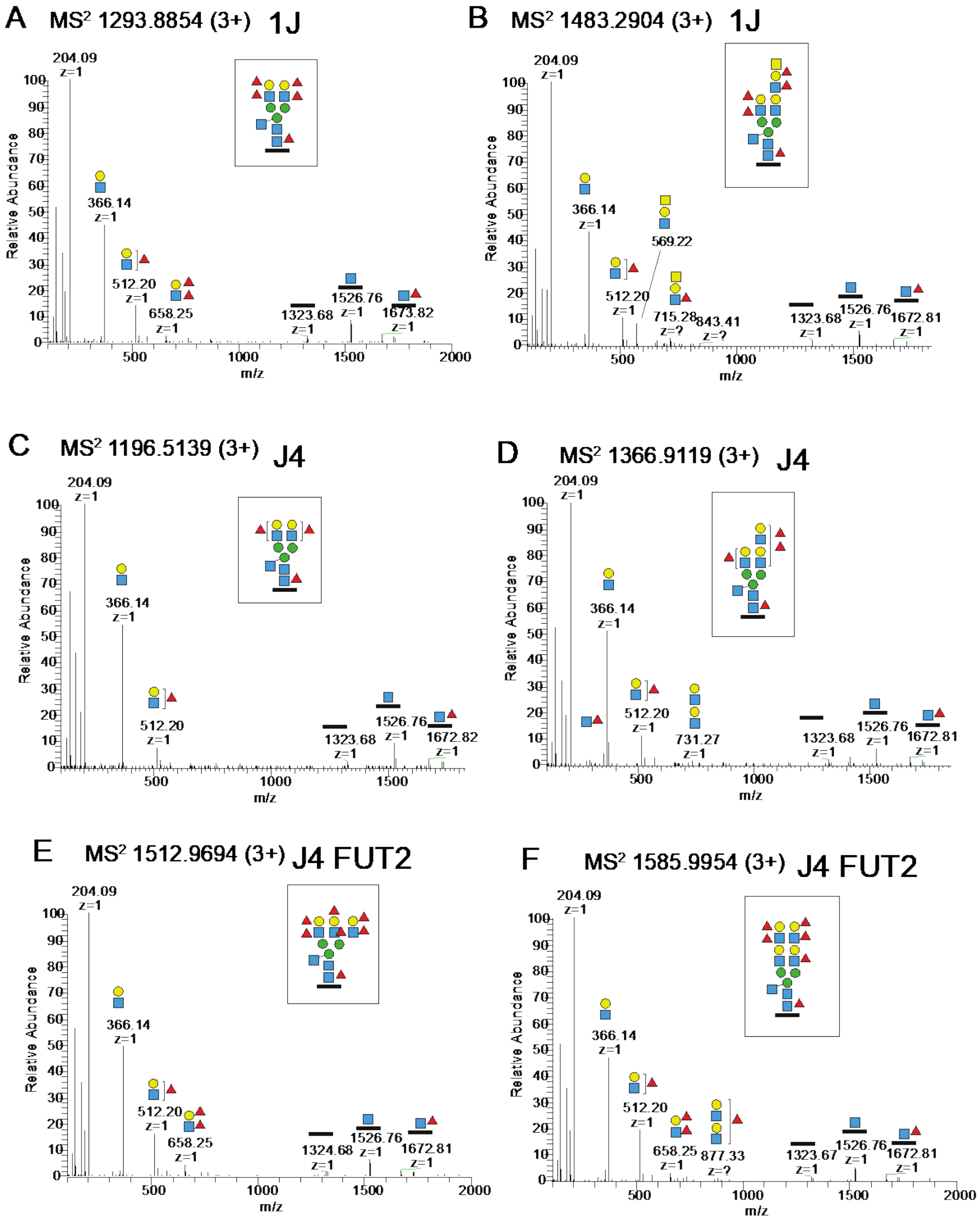

Figure S10. **Examples of MS² spectra of tetraspanin-8 (TSN8)** ***N*****-glycopeptides** from (**A-B**) 1J; (**C-D**) J4; and (**E-F**) J4FUT2 HIEs.

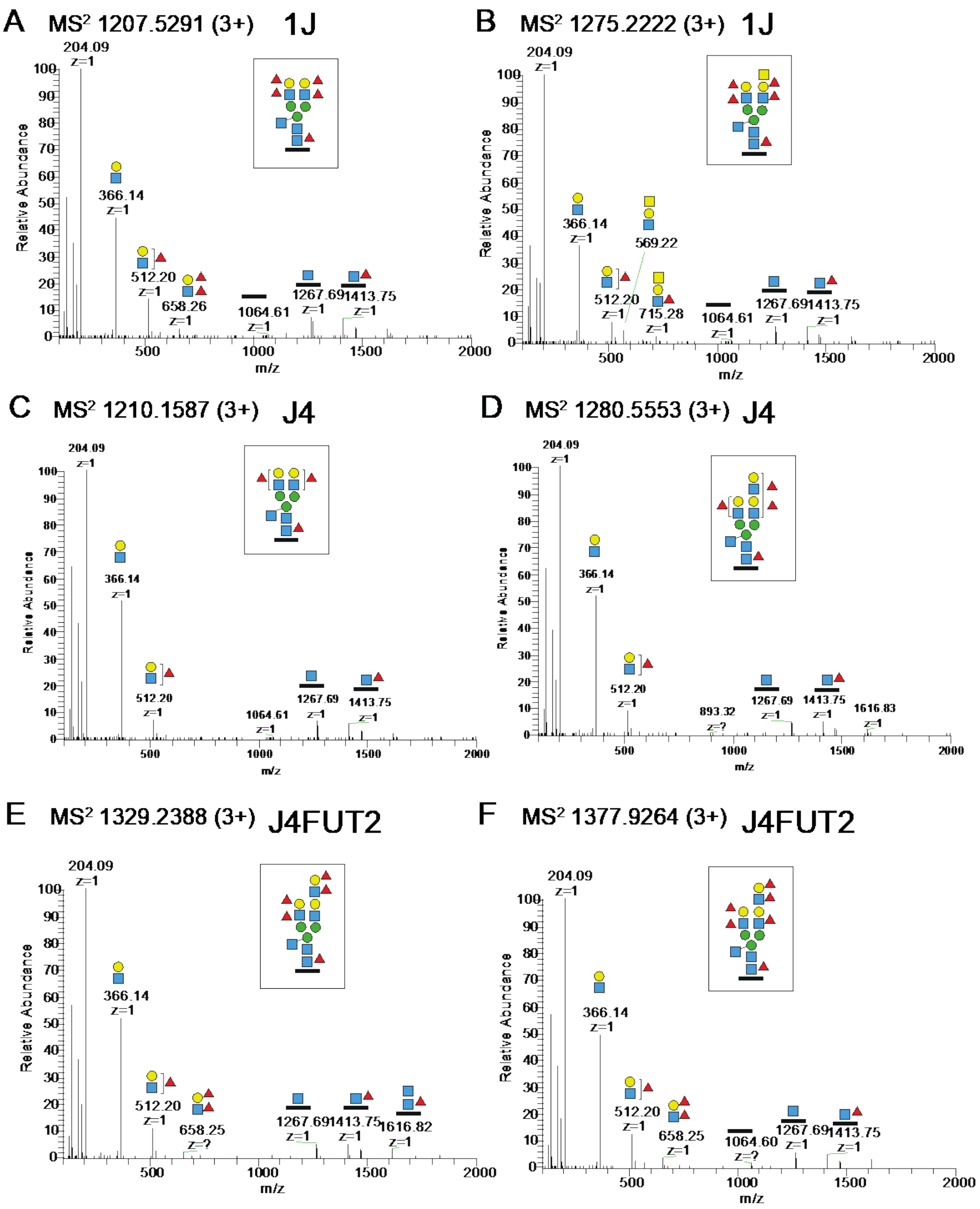

Figure S11. Examples of MS² spectra of carcinoembryonic antigen-related cell adhesion molecule 5 (CEAM5) glycopeptides containing the Asn-204/560 glycosite for (**A-B**) 1J; (**C-D**) J4; and (**E-F**) J4FUT2 HIEs.

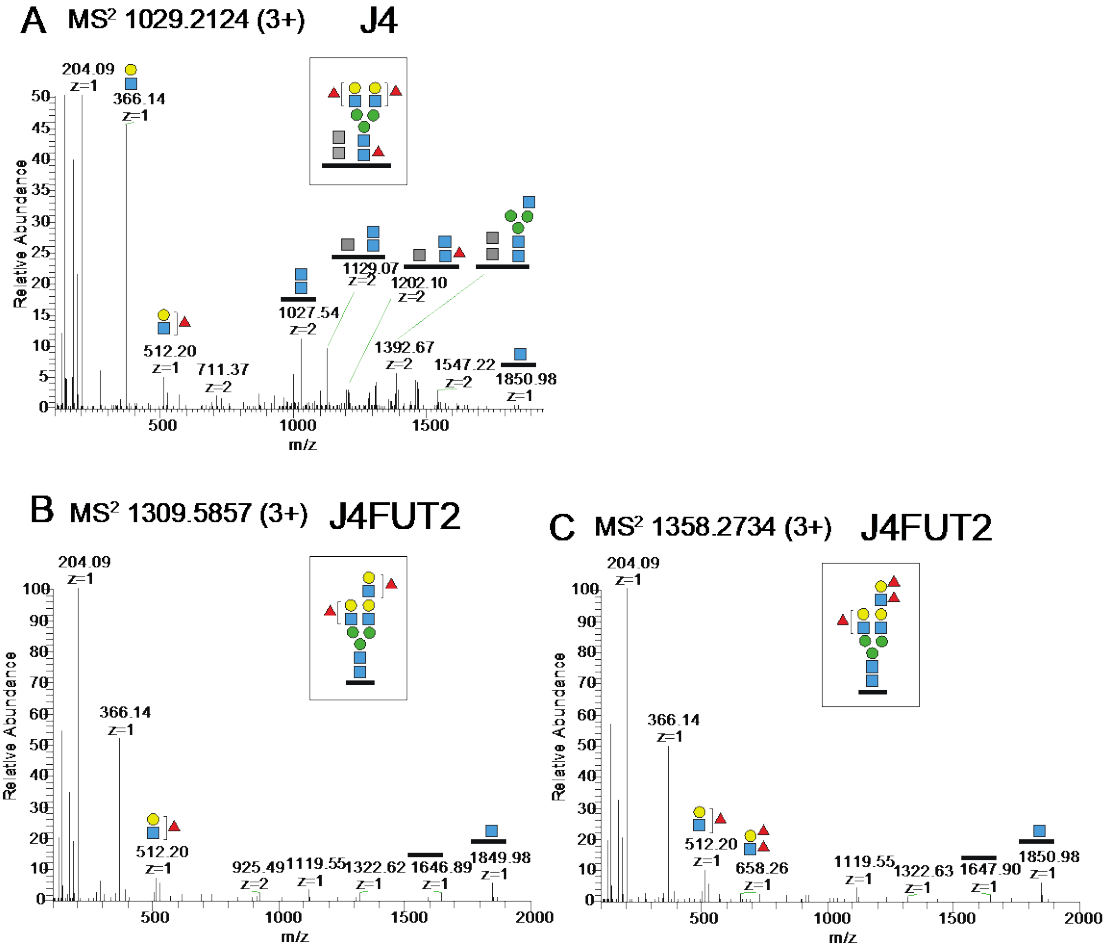

Figure S12. **Examples of MS^2 spectra of sucrase/isomaltase (SUIS) glycopeptides** from (**A**) J4; (**B-C**) J4FUT2 HIEs. The peptide sequence is YIIILDPAISGNETK including the Asn-1303 glycosylation site.

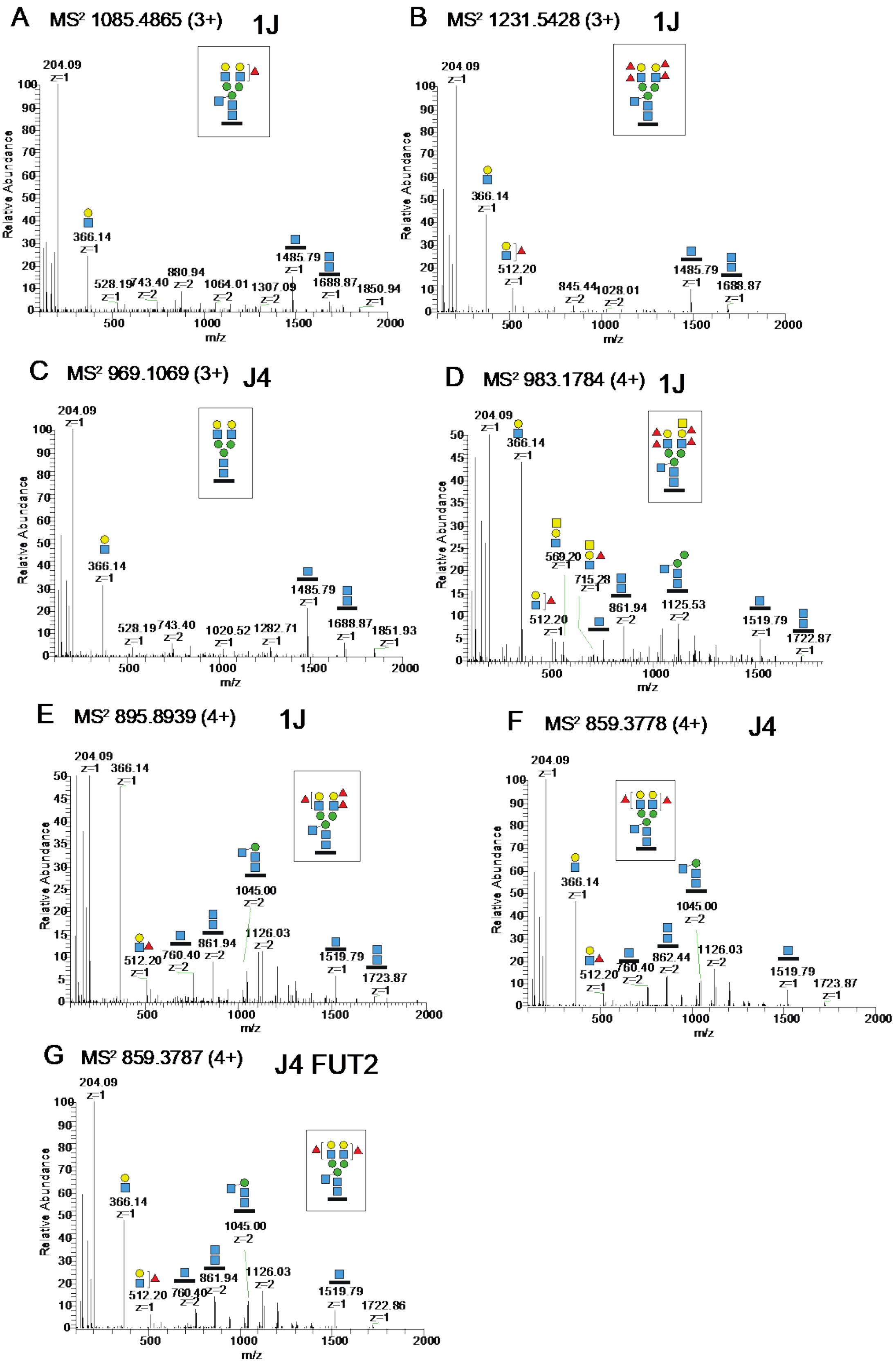

Figure S13. **Examples of MS² spectra of aminopeptidase-N (AMPN) glycopeptides**. The peptide sequence is AEFNITLIHPK including the Asn-234 glycosylation site for (**A-B**) 1J and (**C**) J4 HIEs The peptide sequence is KLNYTLSQGHR including the Asn-128 glycosylation site for (**D-E**) 1J, (**F**) J4 and (**G**) J4FUT2 HIEs.

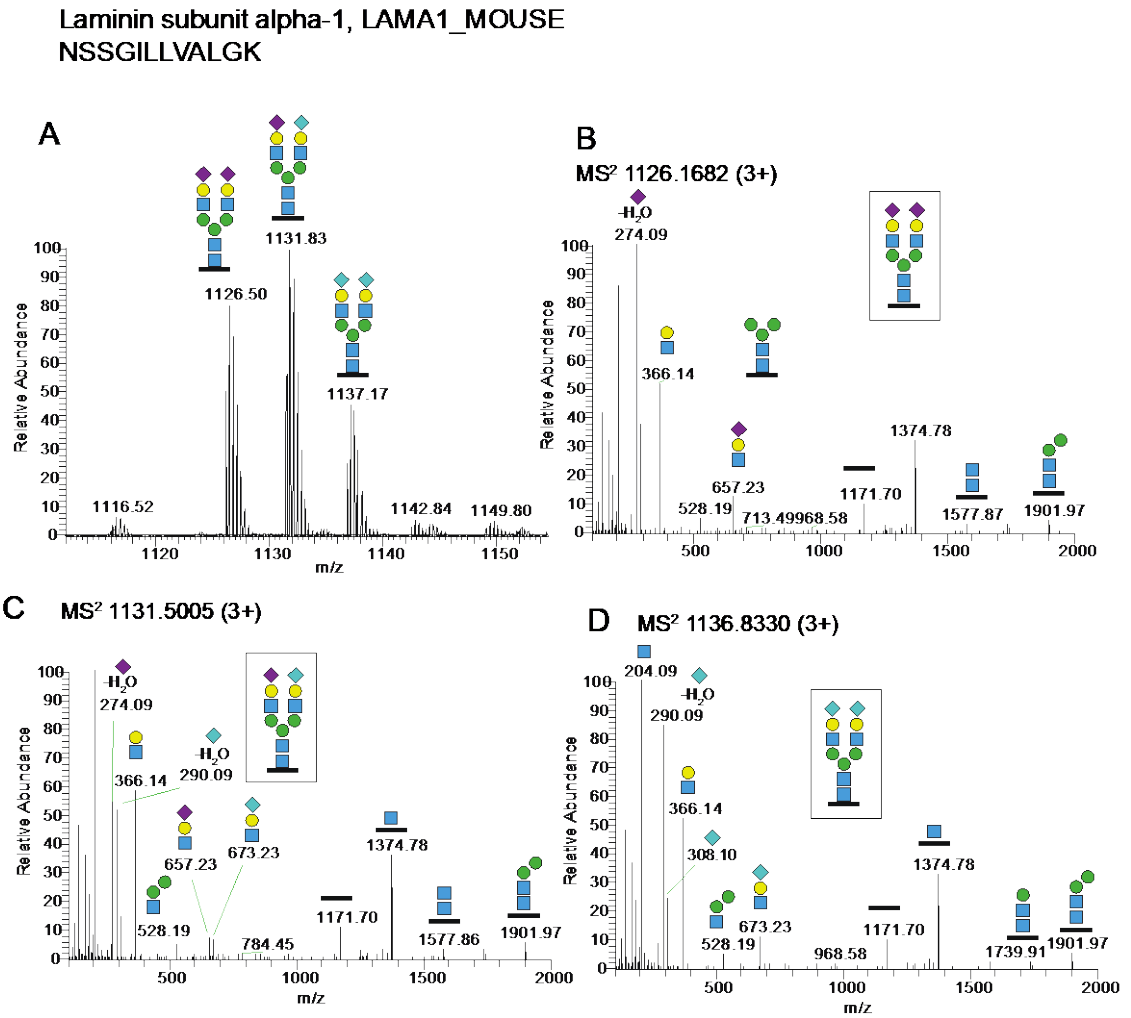

Figure S14. MS^2 identification of sialylated *N*-glycopeptides originating from Matrigel glycoproteins in a J2 HIE preparation. (**A**) MS^1 spectrum of several sialic acid glycoforms of glycopeptides with the amino acid sequence NSSGILLVALGK from laminin subunit alpha-1 (LAMA1_MOUSE). (**B**) MS^2 spectrum of the $(Neu5Ac)_2$ glycoform, (**C**) of the Neu5Ac-Neu5Gc glycoform(s) and (**D**) of the $(Neu5Gc)_2$ glycoform.

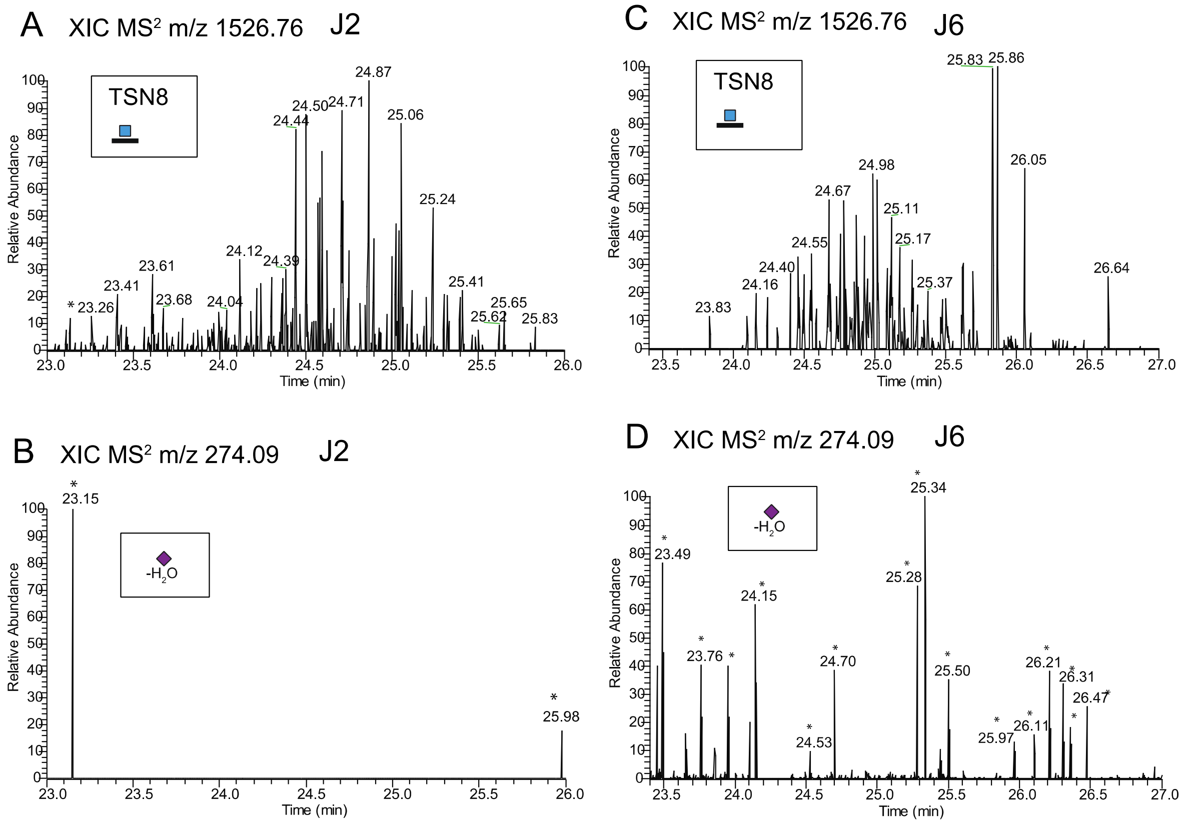

Figure S15. XIC MS^2 assay to investigate the presence of Neu5Ac glycoforms among the TSN8 glycopeptides of the J2 and J6 HIE preparations. (**A**) XIC MS^2 at *m/z* 1526.76 corresponding to the diagnostic peptide+HexNAc ion of the TSN8 glycopeptides for a J2 HIE sample; and (**B**) at *m/z* 274.09 corresponding to the Neu5Ac-H_2O oxonium ion. (**C** and **D**) the corresponding XICs for a J6 HIE sample. *Ion not associated with TSN8. No Neu5Ac oxonium ions were found to be associated with the MS^2 spectra of TSN8 glycopeptides, or any other of the described glycopeptides carrying HBGAs.

The *m/z* 274.09 peak at 23.49 min for J6 is from a glycopeptide with NLSEIK sequence from laminin subunit alpha-1 (mouse). The *m/z* 274.09 peaks at 23.76 min and 26.47 min for J6 are from glycopeptides with NISQDLEK sequence from laminin subunit gamma-1 (mouse and human). The peak at 24.15 min for J6 is from a glycopeptide with RIPAINR sequence from laminin subunit gamma-1 (mouse). The peaks at 24.70 min; 25.28 min; 25.34 and 25.50 min for J6 are from glycopeptides with ELAAAANESAVK sequence from laminin subunit alpha-1 (mouse), (Supplementary Table S4).

Table S2. Protein characteristics of 23 of 24 glycoproteins carrying fucosylated N-glycans identified in the HIE cultures. Core fucose and HBGA glycoforms (with or without core fucose) are included. (Laminin subunit gamma-1 (P11047) was excluded due to its Matrigel origin.)

Protein ID| Abbreviation|Gene	Protein Name	Mass (kDa)	Protein type	Cellular location[a]	Function
Q9HDC9| APMAP_HUMAN|APMAP	Adipocyte plasma membrane-associated protein	46	type II	PM	Arylesterase activity, strictosidine synthase activity, biosynthetic process
**P15144| AMPN_HUMAN|ANPEP**	**Aminopeptidase N**	**110**	**type II**	**PM**	**Hydrolyses N-terminal amino acids from proteins, prohormones and cytokines, receptor for human Coronavirus 229E and also mediates human CMV infection.**
P16278| BGAL_HUMAN|GLB1	Beta-galactosidase	76	soluble	lysosome	Cleaves off terminal non reducing beta-galactose residues of gangliosides, glycoprotein and proteoglycans
Q12864| CAD17_HUMAN|CDH17	Cadherin-17	92	type I	PM	Connecting cells, intestinal peptide transport
P13688| CEAM1_HUMAN|CEACAM1	Carcinoembryonic antigen-related cell adhesion molecule 1	58	type I	PM	Cell adhesion
**P06731| CEAM5_HUMAN|CEACAM5**	**Carcinoembryonic antigen-related cell adhesion molecule 5**	**180**	**GPI-anchor**	**PM**	**Plays a role in cell adhesion and tumor progression and is also defined as a receptor for E. coli Dr adhesion**
P07339| CATD_HUMAN|CTSD	Cathepsin D	45	soluble	lysosome, extracellular space	Acidic protease
P14625| ENPL_HUMAN|HSP90B1	Endoplasmin	92	lumen	ER	Endothelial reticulum associated degradation of proteins
Q08380| LG3BP_HUMAN|LGALS3BP	Galectin-3-binding protein	65	secreted	secretory vesicles, body fluids	Cell adhesion
P04062<br>GBA1_HUMAN|GBA1	Lysosomal acid glucosylceramidase	60	membrane	lysosome	Catalyzes the hydrolysis of glucosylceramides into free ceramides and glucose

P10253| LYAG_HUMAN|GAA	Lysosomal alpha-glucosidase	105	secreted/ membrane	lysosome	Hydrolyses glycogen, preferentially alpha1,4-linkages but also alpha1,6-linkages
P11279| LAMP1_HUMAN|LAMP1	Lysosome-associated membrane glycoprotein 1	45	type I	endosome, lysosome, PM, secreted	Lassavirus entry into the cells
P13473| LAMP2_HUMAN|LAMP2	Lysosome-associated membrane glycoprotein 2	45	type I	lysosome, endosome, PM	Plays a role in chaperone mediated autophagy, stimulating lysosomal degradation of proteins and is required for efficient MHCII-mediated presentation of exogenous antigens
Q9H3R2 MUC13_HUMAN|MUC13	Mucin-13	55	type I	PM, secreted	May play a role in cell signaling
Q6UX06 OLFM4_HUMAN|OLFM4	Olfactomedin-4	57	screted	secreted	Cell adhesion
P01833| PIGR_HUMAN|PIGR	Polymeric immunoglobulin receptor	83	type I	PM, secreted	Binds polymeric IgA and IgM on the basolateral surfaces of epithelial cells and then the complex is incorporated and transported across the cell and secreted on the apical surface
P07602 SAP_HUMAN|PSAP	Prosaposin	58	secreted	lysosome	Myelinotropic and neurotrophic factor
Q15293 RCN1_HUMAN|RCN1	Reticulocalbin-1	39	soluble	ER lumen	May regulate calcium-dependent activities in the endoplasmic reticulum lumen or post-ER compartment
**P14410| SUIS_HUMAN|SI**	**Sucrase-isomaltase, intestinal**	**209**	**type II**	**PM**	**Hydrolytic activity towards starch, sucrose (Glc*f*a1-2Fru*p*) and isomaltose (Glc*f*a1-6Glc*f*)**
**P19075| TSN8_HUMAN|TSPAN8**	**Tetraspanin-8**	**26**	**multi-pass**	**PM**	**Integrin binding, negative regulation of blood coagulation, regulation of gene expression, spermatogenesis**
Q9HD45| TM9S3_HUMAN|TM9SF3	Transmembrane 9 superfamily member 3	68	multi-pass	membrane	Protein localization to membrane
O14773 TPP1_HUMAN|TPP1	Tripeptidyl-peptidase 1	61	soluble	lysosome	Lysosomal serine protease
Q9HAW8 UD110_HUMAN|UGT1A10	UDP-glucuronosyltransferase 1A10	60	single-pass	ER membrane	Catalyzes phase II biotransformation reactions in which lipophilic substrates are conjugated with glucuronic acid to increase the metabolite's water solubility, thereby facilitating excretion into either the urine or bile

[a]PM=plasma membrane, ER=endoplasmic reticulum

Table S3. Expression of fucosyltransferase, sialidase (neuraminidase) and sialyltransferase mRNAs in J2 HIEs.

Gene Symbol	Transferase Activity	Absolute Expression[a]	Relative Expression[b]
FUT1	Alpha-(1,2)	112	0.02
FUT2	Alpha-(1,2)	6587	1.00
FUT3	Alpha-(1,3/1,4)	9276	1.41
FUT4	Alpha-(1,3)	3411	0.52
FUT5	Alpha-(1,3/1,4)	--	ND
FUT6	Alpha-(1,3)	2485	0.38
FUT7	Alpha-(1,3)	--	ND
FUT8	Alpha-(1,6)	2534	0.38
FUT9	Alpha-(1,3)	47	0.01
FUT10	Alpha-(1,3) putative	715	0.11
FUT11	Alpha-(1,3) putative	1568	0.24

Gene Symbol	Sialidase Activity	Absolute Expression[a]	Relative Expression[b]
NEU1	Alpha-(2,3/2,6)	12108	1.8
NEU2	Alpha-(2,3)	2	0.00
NEU3	Alpha-(2,3/2,8)	192	0.03
NEU4	Alpha-(2,3/2,8)	229	0.03

Gene Symbol	Transferase Activity	Absolute Expression[a]	Relative Expression[b]
ST3GAL1	Alpha-(2,3)	1844	1.00
ST3GAL2	Alpha-(2,3)	434	0.24
ST3GAL3	Alpha-(2,3)	60	0.03
ST3GAL4	Alpha-(2,3)	--	ND
ST3GAL5	Alpha-(2,3)	29	0.02
ST3GAL6	Alpha-(2,3)	20	0.01
ST6GALNAC1	Alpha-(2,6)	4002	2.17
ST6GALNAC2	Alpha-(2,6)	57	0.03
ST6GALNAC3	Alpha-(2,6)	1	0.00
ST6GALNAC4	Alpha-(2,6)	1378	0.75
ST6GALNAC5	Alpha-(2,6)	--	ND
ST6GALNAC6	Alpha-(2,6)	506	0.27
ST6GAL1	Alpha-(2,6)	--	ND
ST6GAL2	Alpha-(2,6)	--	ND
ST8SIA1	Alpha-(2,8)	25	0.01
ST8SIA2	Alpha-(2,8)	--	ND
ST8SIA3	Alpha-(2,8)	180	0.10
ST8SIA4	Alpha-(2,8)	--	ND
ST8SIA5	Alpha-(2,8)	--	ND
ST8SIA6	Alpha-(2,8)	650	0.35

[a]Counts per million from J2 media. The RNA-seq data files and raw count matrix files are deposited in the Gene Expression Omnibus (GSE150918).
[b]For comparison the expression data are normalized to the *FUT2* and *ST3GAL1* expression levels, respectively. ND=not detected

Table S4. Sialic acid Neu5Ac and Neu5Gc N-glycopeptide identities from HIE preparations. Byonic searches were done both with the human and mouse databases, and the protein abbreviations include the HUMAN and MOUSE IDs.

Protein ID|Abbreviation	Protein Name	HIEs containing glycopeptides with this peptide sequence	Peptide Sequence	*Sialic acid type*[a]	Glycosite Asn N:o
Q9Y4L1|HYOU1_HUMAN	Hypoxia up-regulated protein 1	J2	R.VFGSQNLTTVK.L	Neu5Gc	515
Q9JKR6|HYOU1_MOUSE	Hypoxia up-regulated protein 1	J2	R.VFGSQNLTTVK.L	Neu5Gc	515
P19137|LAMA1_MOUSE	Laminin subunit alpha-1	1J, J2, J4, J4FUT2, J6	R.QISINNTAVMQR.L	Neu5Ac, Neu5Gc	561
P19137|LAMA1_MOUSE	Laminin subunit alpha-1	1J, J2, J4, J4FUT2, J6[b]	R.IANISMEVGR.K	Neu5Ac, Neu5Gc	1344
P19137|LAMA1_MOUSE	Laminin subunit alpha-1	1J, J2, J4, J4FUT2, J6	K.EANSLLSNHSEK.L	Neu5Ac, Neu5Gc	1763
P19137|LAMA1_MOUSE	Laminin subunit alpha-1	1J, J2, J4, J4FUT2	R.VQEEQNVTSELIAK.G	Neu5Ac, Neu5Gc	1812
P19137|LAMA1_MOUSE	Laminin subunit alpha-1	1J, J2, J4, J4FUT2	K.TANKTDLISESLASR.G	Neu5Ac, Neu5Gc	1936
P19137|LAMA1_MOUSE	Laminin subunit alpha-1	1J, J2, J4, J4FUT2, J6	R.ELAAAANESAVK.T	Neu5Ac, Neu5Gc	2027
P25391|LAMA1_HUMAN	Laminin subunit alpha-1	1J, J2, J4, J4FUT2, J6	R.NLSEIK.L	Neu5Ac, Neu5Gc	2098
P19137|LAMA1_MOUSE	Laminin subunit alpha-1	1J, J2, J4, J4FUT2, J6	R.NLSEIK.L	Neu5Ac, Neu5Gc	2106
P19137|LAMA1_MOUSE	Laminin subunit alpha-1	1J, J4	K.VLDINNSTLMFVGGLGGQIK.K	Neu5Ac, Neu5Gc	2251/2252
P19137|LAMA1_MOUSE	Laminin subunit alpha-1	1J, J2, J4, J4FUT2, J6	K.NSSGILLVALGK.D	Neu5Ac, Neu5Gc	2526
P19137|LAMA1_MOUSE	Laminin subunit alpha-1	1J, J4	R.KAFMTVDGQESPSVTVVGNATTLDVER.K	Neu5Ac, Neu5Gc	2835
P02469|LAMB1_MOUSE	Laminin subunit beta-1	1J, J4	K.LTDTASQSNSTAGELGALQAEAESLDK.T	Neu5Ac	1279
P02469|LAMB1_MOUSE	Laminin subunit beta-1	1J, J2, J4, J4FUT2, J6	R.VNASTTDPNSTVEQSALTR.D	Neu5Ac, Neu5Gc	1336
F8VQJ3|F8VQJ3_MOUSE	Laminin subunit gamma-1	1J, J2, J4, J4FUT2, J6	K.LLNNLTSIK.I	Neu5Ac, Neu5Gc	648
P11047|LAMC1_HUMAN	Laminin subunit gamma-1	1J, J2, J4, J4FUT2, J6	K.LLNNLTSIK.I	Neu5Ac, Neu5Gc	650

F8VQJ3|F8VQJ3_MOUSE	Laminin subunit gamma-1	1J, J2, J4, J4FUT2, J6	R.VNSSLHSQISR.L	Neu5Ac, Neu5Gc	1104
F8VQJ3|F8VQJ3_MOUSE	Laminin subunit gamma-1	1J, J2, J4, J6	K.TANETSAEAYNLLLR.T	Neu5Ac, Neu5Gc	1203
P11047|LAMC1_HUMAN	Laminin subunit gamma-1	1J, J2, J4, J6	K.TANDTSTEAYNLLLR.T	Neu5Ac, Neu5Gc	1205
F8VQJ3|F8VQJ3_MOUSE	Laminin subunit gamma-1	1J, J2, J4	R.TLAGENQTALEIEELNR.K	Neu5Ac, Neu5Gc	1221
F8VQJ3|F8VQJ3_MOUSE	Laminin subunit gamma-1	1J, J2, J4, J4FUT2, J6	K.NISQDLEK.Q	Neu5Ac, Neu5Gc	1239
P11047|LAMC1_HUMAN	Laminin subunit gamma-1	1J, J2, J4, J4FUT2, J6	K.NISQDLEK.Q	Neu5Ac, Ne5uGc	1241
F8VQJ3|F8VQJ3_MOUSE	Laminin subunit gamma-1	1J, J2, J4, J4FUT2, J6	R.RIPAINR.T	Neu5Ac, Neu5Gc	1393

[a]Neu5Ac=N-acetylneuraminic acid, Neu5Gc=N-glycolylneuraminic acid, [b]For J6 this peptide sequence was R.IANISMEVGRK.A
